# Reversibly Cross-Linked Asymmetric Hybrid Open-Polysilsesquioxane Films Enhancing Clotrimazole Bioavailability and Anti-*Candida* Mature Biofilm Activity for Vaginal Therapy

**DOI:** 10.1021/acsami.5c12791

**Published:** 2025-10-07

**Authors:** Marta Madej-Gajewska, Tomasz Janek, Monika Gosecka, Mateusz Gosecki, Malgorzata Urbaniak, Ewelina Wielgus, Łukasz John

**Affiliations:** † Faculty of Chemistry, University of Wrocław, 14 F. Joliot-Curie, Wrocław 50-383, Poland; ‡ Department of Biotechnology and Food Microbiology, 56641 Wrocław University of Environmental and Life Sciences, 37 Chełmonskiego, Wrocław 51-630, Poland; § Centre of Molecular and Macromolecular Studies, Polish Academy of Sciences, Sienkiewicza 112, Łódź 90-363, Poland

**Keywords:** network, film formulation, antifungal intravaginal therapy, boronic ester, incompletely condensed POSS cage, cross-linkable open-POSS cage, swelling ratio, *Candida* biofilm inhibition

## Abstract

Vulvovaginal candidiasis, primarily caused by *Candida albicans*, presents a significant therapeutic challenge due to fungal biofilm formation and the poor aqueous solubility of azole antifungals like clotrimazole, CLT. Films are increasingly favored as antimicrobial drug carriers due to their capacity to provide prolonged vaginal retention, extended shelf life, and simplified storage compared to traditional drug forms. Current film formulations, however, often suffer from nonuniform drug distribution, uncontrolled drug release, and compromised structural integrity. To overcome these limitations, we developed novel, water-swellable polymeric networks designed for enhanced clotrimazole bioavailability and potent anti-*Candida* biofilm activity. Our strategy involved the reversible cross-linking of unique asymmetric open-Polyhedral Oligomeric Silsesquioxane (POSS) cages, functionalized with both hydrophobic, i.e., phenyl (**IC-POSS** ^**Ph**^) or isobutyl (**IC-POSS** ^**iBu**^) groups and bearing hydrophilic 1,2-diol moieties, with poly­(dimethylacrylamide-2-acrylamidephenylboronic acid) (P­(DMAM-2-AAPBA)) copolymers. We tailored the copolymer composition to achieve precise control over the network cross-linking density. Comprehensive characterization, including ^11^B NMR spectroscopy, differential scanning calorimetry, rheology, and SEM-EDS (scanning electron microscopy-energy dispersive X-ray spectroscopy), elucidated the structure–property relationships. We demonstrated that **IC-POSS** ^**Ph**^ cages intrinsically prevent CLT crystallization, likely via π–π-stacking interactions, facilitating homogeneous drug distribution. Conversely, while **IC-POSS** ^**iBu**^ cages showed less inherent drug compatibility, the P­(DMAM-2-AAPBA) copolymers were crucial for achieving uniform CLT dispersion within these networks. Our studies revealed that higher 2-AAPBA content in the copolymer increased network cross-linking density, leading to slower drug release. Moreover, π–π interactions between **IC-POSS** ^**Ph**^ cages in the networks contributed to a reduced swelling capacity and evidently slower drug release. Crucially, biological evaluations confirmed that these CLT-loaded polymeric films significantly enhanced antifungal efficacy against both planktonic *C. albicans* strains (ATCC 10231 and SC5314) and mature *Candida* biofilms, outperforming free CLT. This superior performance is attributed to the networks’ ability to maintain CLT in the molecular state and enable its controlled release, thereby improving its bioavailability at the target site. The elaborated films also exhibited good cytocompatibility. This work highlights how subtle structural modifications in network components are crucial to achieving desired biological functions, representing a promising advance for antifungal drug delivery and, in general, hydrophobic drug carriers in various biomedical applications.

## Graphical abstract

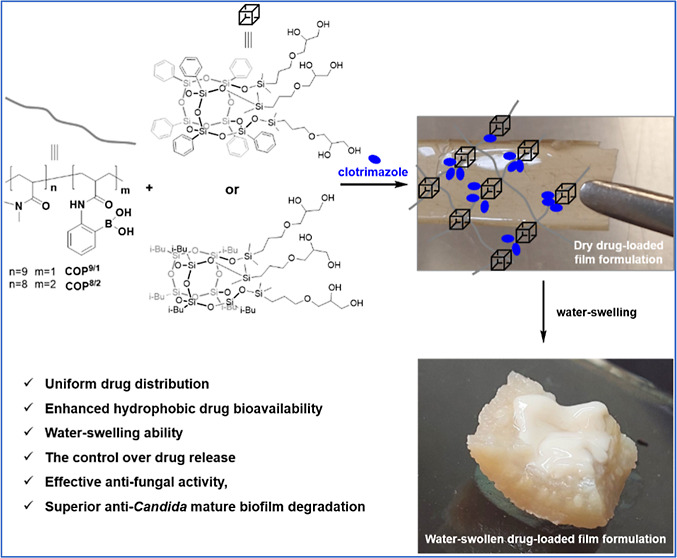

## 1 Introduction

Vulvovaginal candidiasis is the second most commonly reported vaginal disease in the world caused by fungi, mainly *Candida albicans*, constituting one-third of all cases of vulvovaginitis in women of reproductive age.^1,2^ Vulvovaginal candidiasis occurs when *Candida* fungi penetrate the surface of the vaginal mucosa and cause an inflammatory reaction. The inflammatory reaction causes profuse, thick vaginal discharge and vaginal irritation, abrasions, painful urination, itching, burning, dyspareunia, and other symptoms.^3^ Furthermore, *C. albicans*’ ability to form biofilms on vaginal epithelium^4^ in vivo presents a significant challenge to effective antifungal therapies.

The main factors contributing to acute vulvovaginal candidiasis include the use of estrogens, a broad spectrum of antibiotics, and lifestyle diseases, such as obesity and diabetes.^5,6^ The therapy for vulvovaginal candidiasis primarily relies on antifungal azole-based compounds such as clotrimazole, fluconazole, itraconazole, or voriconazole, all of which exhibit very low solubility in aqueous solutions, including physiological conditions. The most common treatment for candidiasis involves clotrimazole-loaded therapeutics, typically administered as tablets or as globules. However, due to clotrimazole’s low water solubility (0.49 μg/mL),^7^ and uncontrolled release from commercial formulations, frequent dosing is needed to maintain efficacy and prevent recurrent or azole-resistant candidiasis.^8–10^ To address the limitations of commercially available therapeutics, efforts are being made to develop clotrimazole-loaded delivery systems that exhibit a yield stress at the physiological conditions of the vagina, such as hydrated formulations, i.e., hydrogels,^11–13^ Bingham fluids,^14^ or solid polymeric films.^15^ Film-type formulations are preferred over hydrated carriers for poorly soluble drugs due to better stability and storage, but ensuring uniform drug distribution remains a challenge, affecting structural integrity.^16^ This poor integrity leads to premature drug release (within 8 h),^16^ necessitating frequent administration. Until now, the construction of polymer films has not been explored well and is mainly based on such polymers as HPMC (hydroxypropyl methylcellulose), PVA (poly­(vinyl alcohol)), PEO (poly­(ethylene oxide)), and PLGA (poly­(lactic-*co*-glycolic acid)).^16^ The construction of films of desired characteristics including flexibility, homogeneous distribution of bioactive compounds, and their permeability, requires the usage of additional compounds like surfactants,^17,18^ plasticizers,^19^ or permeation enhancers^20^ (e.g., DMSO, dimethyl sulfoxide). The manufacturing process necessitates the use of at least two solvents to fully solubilize all film components, including the drug.^21^

To overcome current drawbacks in drug-loaded film formation, in this work, we present a simple strategy for the construction of clotrimazole-loaded films ensuring homogeneous drug distribution. This approach utilizes asymmetric, incompletely condensed polyhedral oligomeric silsesquioxane (open-POSS) cages, which have distinct hydrophilic and hydrophobic sites. The idea of the usage of POSS cages for the formation of drug-loaded films was dictated by their ability to modulate surface hydrophobicity and improved compatibility with various polymer matrices^22–26^ as a pivotal feature for the enhancement of bioavailability and transport of poorly water-soluble drugs. Novel cages with hydrophobic (isobutyl, iBu/phenyl, and Ph) and hydrophilic (1,2-diol) groups were synthesized to boost clotrimazole bioavailability and enable water-swellable, cross-linked films with boronic acid-containing copolymers. For the formation of drug-loaded films, noncytotoxic dimethylacrylamide copolymers bearing different 2-acrylamidephenylboronic acid contents were used to obtain networks differing in the cross-linking density crucial for precise drug release control. Such the construction of elaborated films can ensure the delivery of drugs in the molecular state with effective activity against *C. albicans* ATCC 10231 and SC5314 species, and what is of great importance, superior degradation ability of mature *Candida*-based biofilms, typical for vulvovaginal candidiasis.

In this study, we investigate how the structural features of asymmetric-POSS cages, specifically the type of hydrophobic groups (phenyl or isobutyl) and the copolymer composition (molar ratio of 2-acrylamidephenylboronic acid to dimethylacrylamide), influence polymer chain mobility, water-swelling capacity, drug release profiles, and antifungal efficacy of the resulting films. This work highlights how subtle structural changes in the network components can significantly impact biological function. The developed film formulation strategy offers promise not only for antifungal therapy but also for delivery of hydrophobic drugs in broader biomedical applications.

## 2 Experimental Section

### 2.1 Materials

All the chemicals were obtained from commercial sources and used without further purification: trisilanolisobutyl-POSS (Hybrid Plastics Inc.), trisilanolphenyl-POSS (Hybrid Plastics Inc.), chlorodimethylsilane (≥97.5%; Sigma-Aldrich), 3-allyloxy-1,2-propanediol (99%; Sigma-Aldrich), and Karstedt catalyst (platinum(0)-1,3-divinyl-1,1,3,3-tetramethyldisiloxane complex solution) (in xylene, Pt ∼ 2%, Sigma-Aldrich). Anhydrous magnesium sulfate (MgSO_4_, ≥99%) was obtained from EuroChem and used without further purification. Solvents for synthesis: THF (tetrahydrofuran), dichloromethane (CH_2_Cl_2_), and hexane (C_6_H_14_) were purified using the Solvent Purification System (Inert, PureSolv EN 1–7 Base). Methanol was obtained from EuroChem and used without further purification. The catalyst was removed after the reaction by filtration through a Celite 545 (Carl Roth) pad.

Comonomer 2-acrylamidephenylboronic acid pinacol ester, 2-AAPBAE, was synthesized according to the procedure reported in ref 27. The α,α′-azobis­(isobutyronitrile), AIBN (Fluka), was recrystallized from methanol. Clotrimazole (CLT; Sigma-Aldrich) was used as received. Dialysis tubes (SnakeSkin TM 3.5 K MWCO) were purchased from Thermo Fisher Scientific.

The solvent used during the NMR measurements performed for the analysis of open-POSS cages and their precursors was chloroform-*d*. Spectra were referenced based on the residual solvent signal, using the following values: 7.26 ppm for ^1^H NMR spectra and 77.00 ppm for ^13^C NMR spectra. The ^1^H NMR spectra for copolymers of dimethylacrylamide and 2-acrylamidophenylboronic acid were recorded in D_2_O, taking the signal of the residual solvent at 4.79 ppm. Chemical shift values for ^29^Si NMR spectra were referenced to TMS or DSS (2,2-dimethylsilapentane-5-sulphonate).

### 2.2 Syntheses of Incompletely Condensed Cage-Like Silsesquioxane Hybrids

Synthetic protocols for hepta­(isobutyl)­tris­(dimethylsiloxy)-POSS and hepta­(phenyl)­tris­(dimethylsiloxy)-POSS are based on a slightly modified literature procedure.^28^ These syntheses were performed under an inert atmosphere (N_2_).

### 2.3 Synthesis of Hepta­(isobutyl)­tris­(dimethylsiloxy)-POSS

A Schlenk flask immersed in an ice bath was charged with dry hexane (50 mL) and triethylamine (0.0395 mol, 5.5 mL). Trisilanolisobutyl-POSS (0.0126 mol, 10 g) was then added, followed by the dropwise addition of chlorodimethylsilane (0.0388 mol, 4.34 mL). The reaction mixture became cloudy, indicating the formation of triethylammonium chloride. After being stirred at 0 °C for 1 h, the mixture was gradually brought to room temperature and stirred for an additional 23 h. Upon completion, the reaction mixture was filtered to remove the solid byproduct, and the filtrate was concentrated using a rotary evaporator. The crude product was recrystallized from methanol, collected by filtration, washed three times with methanol, and dried under a vacuum. Yield: 85%. ^1^H NMR (500 MHz, CDCl_3_): δ 4.75 (p, *J*_H–H_ = 2.7 Hz, 3H), 1.88–1.82 (sep, *J*_H–H_ = 2.8 Hz, 7H), 0.96 (d, *J*_H–H_ = 6.5 Hz, 42H), 0.57 (d, *J*_H–H_ = 7.1 Hz, 14H), 0.23 (d, *J*_H–H_ = 2.8 Hz, 18H). ^13^C NMR (126 MHz, CDCl_3_, 300 K): δ 25.32 (s, 7C), 25.20 (s, 7C), 23.99 (s, 7C), 23.43 (s, 7C), 0.00 (s, 6C). ^29^Si NMR (99 MHz, CDCl_3_, 300 K): δ −5.49 (s, 3Si), −67.12 (s, 1Si), −67.69 (s, 3Si), −68.02 (s, 3Si). IR (cm^–1^): ν_(C–H)_ = 2953 (w), ν_(C–H)_ = 2869 (w), ν_(Si–H)_ = 2140 (w), δ_(C–H)_ = 1465 (w), ν_(Si–O–Si)_ = 1052 (vs), δ_(Si–C)_ = 762 (w), δ_(Si–O–Si)_ = 473 (w). MALDI-MS *m*/*z*: 987.387.

### 2.4 Synthesis of Hepta­(phenyl)­tris­(dimethylsiloxy)-POSS

A Schlenk flask cooled in an ice bath was charged with dry toluene (50 mL) and triethylamine (0.0337 mol, 4.7 mL), followed by the addition of trisilanolphenyl-POSS (0.0107 mol, 10 g). Chlorodimethylsilane (0.0322 mol, 3.58 mL) was then added dropwise. The mixture became cloudy, indicating the formation of triethylammonium chloride. After being stirred at 0 °C for 1 h, the reaction was brought to room temperature and stirred for an additional 23 h. The resulting mixture was filtered to remove the solid byproduct, and the filtrate was concentrated using a rotary evaporator. The crude product was recrystallized from methanol, collected by filtration, washed three times with methanol, and dried under a vacuum. Yield: 87%. ^1^H NMR (500 MHz, CDCl_3_): δ 7.60 (d, *J*_H–H_ = 6.7 Hz, 2H), 7.50–7.39 (m, 6H), 7.35–7.24 (m, 15H), 7.19–7.12 (m, 12H), 4.94 (sep, *J*_H–H_ = 2.8 Hz, 3H), 0.36 (d, *J*_H–H_ = 2.8 Hz, 6H). ^13^C NMR (126 MHz, CDCl_3_, 300 K): δ 133.35 (s, 14C), 131.79 (s, 7C), 129.42 (s, 7C), 126.85 (s, 14C), 0.00 (s, 6C). ^29^Si NMR (99 MHz, CDCl_3_, 300 K): δ −10.25 (s, 3Si), −77.27 (s, 1Si), −77.61 (s, 3Si), −78.24 (s, 3Si). IR (cm^–1^): ν_(O–H)_ = 3075 (w), ν_(C–H)_ = 2963 (w), ν_(Si–H)_ = 2132 (w), δ_(C–H)_ = 1430 (w), ν_(Si–C)_ = 1253 (w), ν_(Si–O–Si)_ = 1053 (vs), δ_Ar_ = 895 (w), δ_(Si–C)_ = 694 (w), δ_(Si–H)_ = 578 (w), δ_(Si–O–Si)_ = 483 (w). MALDI-MS *m*/*z*: 1127.133 {calcd [M + Na]^+^, 1127.14}.

### 2.5 Synthesis of **IC-POSS** ^**iBu**^

In a round-bottom flask equipped with a magnetic stir bar, 0.518 mmol (0.5 g) of hepta­(isobutyl)­tris­(dimethylsiloxy)-POSS, dissolved in tetrahydrofuran, was placed. Then, 0.010 mL of Karstedt’s catalyst was added, and the mixture was stirred at 45 °C for 30 min. After this, 1.71 mmol (0.211 mL) of 3-allyloxy-1,2-propanediol was added. The reaction mixture was heated to 60 °C and stirred continuously for 24 h. After being completed, the solution was cooled to room temperature. It was then passed through a pad of Celite and concentrated using a rotary evaporator. The residue was dissolved in dichloromethane, washed with water, dried over MgSO_4_, and concentrated again using a rotary evaporator. The resulting oil was dried under a vacuum. Yield: 63%. ^1^H NMR (500 MHz, CDCl_3_): δ 3.84 (m, 3H), 3.67 (m, 3H), 3.60 (m, 3H), 3.47 (m, 6H), 3.41 (s, 6H), 2.69 (s, 6H), 1.82 (m, 7H), 1.58 (m, 6H), 0.94 (dd, *J*_H–H_ = 6.6 Hz, 42H), 0.61 (s, 6H), 0.53 (m, 14H), 0.12 (s, 18H). ^13^C NMR (126 MHz, CDCl_3_, 300 K): δ 72.19 (s, 3C), 71.54 (s, 3C), 70.27 (s, 3C), 63.87 (s, 3C), 25.75 (s, 14C), 25.58 (s, 7C), 23.81 (s, 7C), 23.71 (s, 3C), 13.71 (s, 3C), 0.00 (s, 6C). ^29^Si NMR (99 MHz, CDCl_3_, 300 K): δ 9.15 (s, 3Si), −67.25 (s, 1Si), −67.59 (s, 3Si), −67.83 (s, 3Si). IR (cm^–1^): ν_(O–H)_ = 3347 (w), ν_(C–H)_ = 2962 (w), ν_(C–H)_ = 2875 (w), δ_(C–H)_ = 1432 (w), ν_(C–O)_ = 1259 (w), ν_(Si–O–Si)_ = 1052 (vs), δ_(Si–C)_ = 696 (w), δ_(Si–O–Si)_ = 483 (w). MALDI-MS *m*/*z*: 1383.62 {calcd [M + Na]^+^, 1383.59}.

### 2.6 Synthesis of **IC-POSS** ^**Ph**^

In a round-bottom flask equipped with a magnetic stir bar, 0.452 mmol (0.5 g) of hepta­(phenyl)­tris­(dimethylsiloxy)-POSS, dissolved in tetrahydrofuran, was placed. Then, 0.010 mL of Karstedt’s catalyst was added, and the mixture was stirred at 45 °C for 30 min. After this time, 1.49 mmol (0.184 mL) of 3-allyloxy-1,2-propanediol was added. The reaction mixture was heated to 60 °C and stirred continuously for 24 h. Afterward, the solution was cooled to room temperature, passed through a pad of Celite, and concentrated using a rotary evaporator. The residue was dissolved in dichloromethane, washed with water, dried over MgSO_4_, and concentrated again using a rotary evaporator. The resulting oil was dried under a vacuum. Yield: 67%. ^1^H NMR (500 MHz, CDCl_3_): δ 7.38 (dd, *J* = 8.2, 1.4 Hz, 7H), 7.33–7.28 (m, 14H), 7.12 (dt, *J* = 21.5, 7.6 Hz, 14H), 3.81 (q, *J* = 7.1, 6.5 Hz, 3H), 3.66 (dd, *J* = 11.3, 3.7 Hz, 3H), 3.57 (dd, *J* = 11.4, 5.6 Hz, 3H), 3.48–3.35 (m, 12H), 2.81 (s, 3H), 1.83 (s, 3H), 1.68–1.60 (m, 6H), 0.71–0.56 (m, 6H), 0.25 (s, 18H). ^13^C NMR (126 MHz, CDCl_3_, 300 K): δ 133.60 (s, 14C), 132.37 (s, 7C), 130.75 (s, 7C), 127.32 (s, 14C), 74.01 (s, 3H), 71.98 (s, 3H), 70.41 (s, 3C), 63.81 (s, 3C), 22.95 (s, 3C), 13.68 (s, 3C), 0.00 (s, 6H). ^29^Si NMR (99 MHz, CDCl_3_, 300 K): δ 11.93 (s, 3Si), −77.35 (s, 1Si), −77.55 (s, 3Si), −77.97 (s, 3Si). IR (cm^–1^): ν_(O–H)_ = 3375 (w), ν_(C–H)_ = 2956 (w), ν_(C–H)_ = 2870 (w), ν_Ph_ = 1593 (m), δ_(C–H)_ = 1432 (w), ν_(C–O)_ = 1259 (w), ν_(Si–O)_ = 1047 (vs), δ_Ph_ = 840 (w), δ_(Si–C)_ = 696 (w), δ_(Si–O–Si)_ = 483 (w). MALDI-MS *m*/*z*: 1523.38 {calcd [M + Na]^+^, 1523.37}.

### 2.7 Synthesis of Dimethylacrylamide-2-acrylamidephenylboronic Acid Copolymers

#### 2.7.1 Synthesis of Dimethylacrylamide-2-acrylamidephenylboronic Acid Copolymers with 10 mol % Content of 2-AAPBA, P­(DMAM-2-AAPBA), 9:1

The copolymers were prepared by conventional radical polymerization of dimethylacrylamide (4.89 g, 0.0494 mol) and 2-acrylamidephenylboronic acid pinacol ester (1.5 g, 0.00545 mol) dissolved in the mixture of *N*,*N*-dimethylformamide and dioxane (5:1 v/v). AIBN (0.4 μmol, 6.66 mg) was used as an initiator. The copolymerization was performed at 70 °C for 18 h. The polymerization mixture was dialyzed against water using dialysis tubes (1000 cutoff) for 96 h, with the solvent exchanged four times every 24 h to ensure complete hydrolysis of the pinacol boronic ester units and the removal of the released pinacol. The ^1^H NMR spectrum revealed a 9:1 molar composition (Figure S25). Gel permeation chromatography revealed *M*_n_ and dispersity (Table 1).

**Table 1:** Characteristics of Dimethylacrylamide-2-acrylamidephenylboronic Acid Copolymers Obtained via Uncontrolled Radical Copolymerization

P(DMAM-2-AAPBA)	theoretical molar ratio of DMAM to 2-AAPBA	experimental molar ratio of DMAM to 2-AAPBA	*M*_w_	*M*_w_/*M*_n_	*T*_g_, °C
**COP** ^**9/1**^	9:1	9:1	38,000	1.204	99.1
**COP** ^**8/2**^	8:2	8:2	48,000	1.296	93.2

#### 2.7.2 Synthesis of Dimethylacrylamide-2-acrylamidephenylboronic Acid Copolymers with 20 mol % Content of 2-AAPBA, P­(DMAM-2-AAPBA), 8:2

The copolymers were prepared by conventional radical polymerization of dimethylacrylamide (3.62 g, 0.0366 mol) and 2-acrylamidephenylboronic acid pinacol ester (2.5 g, 0.00915 mol) dissolved in the mixture of *N*,*N*-dimethylformamide and dioxane (5:1 v/v). AIBN (0.338 μmol, 5.55 mg) was used as an initiator. The copolymerization was performed at 70 °C for 18 h. The polymerization mixture was dialyzed against water using dialysis tubes (1000 cutoff) for 72 h, with the solvent exchanged four times every 24 h to ensure complete hydrolysis of the pinacol boronic ester units and removal of the released pinacol. ^1^H NMR spectrum revealed 8:2 molar composition (Figure S25). Gel permeation chromatography revealed *M*_n_ and dispersity (Table 1).

^1^H NMR (400 MHz, DMSO-*d*_6_): δ (ppm) 7.65–6.50 (4H, aromatic), 3.20–0.7 ppm (2H, CH_2_, 1H, CH, 6H, 2CH_3_).

### 2.8 Formation of Neat and Clotrimazole-Loaded Films

#### 2.8.1 Formation of Drug-Free Films

The methanolic solution of the open-POSS cage (**IC-POSS** ^**Ph**^ or **IC-POSS** ^**iBu**^) was mixed with the methanolic solution of DMAM-2-AAPBA copolymer (**COP** ^**9/1**^ or **COP** ^**8/2**^) at an equimolar ratio of 1,2-diol groups to 2-AAPBA moieties. The mixture was placed into a Teflon mold and initially evaporated in air at 37 °C, and then in a vacuum oven until the complete removal of methanol, which was confirmed on ^1^H NMR spectra recorded in DMSO-*d*_6_.

#### 2.8.2 Formation of Clotrimazole-Loaded Films

The methanolic solution of the open-POSS cage (**IC-POSS** ^**Ph**^ or **IC-POSS** ^**iBu**^) and clotrimazole was mixed with the methanolic solution of DMAM-2-AAPBA copolymer (**COP** ^**9/1**^ or **COP** ^**8/2**^) at an equimolar ratio of 1,2-diol groups to 2-AAPBA moieties, and a molar ratio of drug to the cage equal to 2:1 or 4:1, respectively. The mixture was placed into a Teflon mold and initially evaporated in air at 37 °C and then in a vacuum oven until the complete removal of methanol, which was confirmed on ^1^H NMR spectra recorded in DMSO-*d*_6_.

### 2.9 Clotrimazole Release Study

A film formulation constructed of the cross-linked **IC-POSS** ^**Ph**^/**IC-POSS** ^**iBu**^ cages via boronic ester linkages containing 0.162 mg of clotrimazole was placed in a dialysis tube (1000 cutoff) immersed in 10 mL of simulated vaginal fluid, SVF, at 37 °C, corresponding to vaginal conditions. For comparison, a suspension of clotrimazole in SVF was also placed in a dialysis tube. Samples were withdrawn at different time intervals. At given time points, 1 mL of the solution was collected and replaced with 1 mL of fresh SVF.

CLT concentrations were determined by an ACQUITY UPLC I-Class chromatography system coupled with a SYNAPT G2-Si mass spectrometer equipped with an electrospray source and a quadrupole time-of-flight mass analyzer (Waters Corp., Milford, MA, USA). An Acquity BEH C18 column (100 × 2.1 mm, 1.7 μm) maintained at 45 °C was used for the chromatographic separation of an analyte. The mobile phase was prepared by mixing 0.1% formic acid (A) and 0.1% formic acid in acetonitrile (B). The elution gradient was: 32% B (0–1.0 min), 32–95% B (1.0–3.0 min), 95–95% B (3.0–3.5 min), 95–32% B (3.5–3.52 min), and 32–32% B (3.52–7.0 min). The flow rate was 0.45 mL/min, and the injection volume was 0.5 μL for CLT.

For mass spectrometric detection, the electrospray source was operated in positive resolution mode. The optimized source parameters were a capillary voltage of 3.0 kV, a cone voltage of 20 V, a desolvation gas flow of 400 L/h at a temperature of 350 °C, nebulizer gas pressure of 6.5 bar, and a source temperature of 100 °C. Mass spectra were recorded over an *m*/*z* range of 100 to 1200. Mass spectrometer conditions were optimized by the direct infusion of a standard solution. The system was controlled using MassLynx software (ver. 4.1), and data processing (peak area integration and construction of the calibration curve) was performed by the TargetLynx software.

The initial stock calibration solution of CLT was created in simulated vaginal fluid. The stock solutions were serially diluted with 0.5 mL of simulated vaginal fluid and 0.5 mL of methanol to obtain working solutions at several concentration levels. The calibration curves were prepared at ten different concentrations of drug solutions and were linear in the concentration range of 0.1 to 20.0 μg/mL for CLT with a correlation coefficient of >0.995.

### 2.10 Biological Investigations

#### 2.10.1 Antifungal Activity

The antifungal activity was evaluated against *C. albicans* ATCC 10231 and SC5314.^29^ The strains were maintained on yeast extract-peptone-dextrose (YPD) agar plates at 4 °C. Before testing, overnight cultures were grown in liquid YPD medium buffered at pH 5.5 with 0.01 M citrate buffer at 30 °C with agitation. Subsequently, 500 μL of the *Candida* suspension, adjusted to a final concentration of 5 × 10^7^ colony-forming units (CFU)/mL, was dispensed into each well of a sterile 48-well microtiter plate. Next, the effect of diol-functionalized open-POSS cages and DMAM-2-AAPBA copolymers (**IC-POSS** ^**a**^ **_COP** ^**b/c**^; where a = Ph or iBu and b/c = 9:1 or 8:2), as well as their CLT-loaded variants (**IC-POSS** ^**a**^ **_COP** ^**b/c**^ **_CLT** ^**d**^; d = 2 or 4), was examined in terms of their impact on *Candida* viability. Free CLT was included as a reference compound for comparative purposes. All polymeric samples, both CLT-loaded and unloaded, were sterilized by UV irradiation for 24 h in a laminar flow cabinet prior to use. Subsequently, the test wells were treated with polymeric constructs (**IC-POSS** ^**a**^ **_COP** ^**b/c**^ **_CLT** ^**d**^) containing 400 μg of CLT per well, along with corresponding amounts of unloaded copolymers (**IC-POSS** ^**a**^ **_COP** ^**b/c**^) serving as polymer controls. Additionally, a solution of CLT (400 μg/well; 800 μg/mL) was used as a reference standard. Untreated wells containing only the *Candida* suspension served as negative growth controls. The plates were incubated at 37 °C for 24 and 48 h. After each incubation period, cell growth was assessed by measuring the optical density at 600 nm (OD_600_) using a BioTek SYNERGY H1 microplate reader (Agilent, Santa Clara, CA, USA) to determine the extent of yeast growth inhibition.

#### 2.10.2 CLT Release Kinetics and Antifungal Activity over Time

To evaluate the sustained antifungal activity of the polymeric systems, a time-dependent CLT release assay was performed over 10 days. Before the experiment, all polymeric constructs (both CLT-loaded and unloaded) were sterilized by UV irradiation for 24 h under aseptic conditions. Films loaded with CLT were incubated in 500 μL of fresh YPD medium buffered at pH 5.5 with 0.01 M citrate buffer at 37 °C. After 24 h, the entire volume of medium (500 μL) was transferred to a fresh 48-well microtiter plate, followed by the addition of 1 μL of an overnight *C. albicans* suspension, resulting in a final concentration of 5 × 10^7^ CFU/mL. The inoculated plate was then incubated at 37 °C for 24 h. *Candida* growth was evaluated by measuring the optical density at 600 nm (OD_600_) using a BioTek SYNERGY H1 microplate reader (Agilent, Santa Clara, CA, USA). Immediately after medium collection, a new aliquot of fresh YPD medium (500 μL) was added to the same polymer samples to initiate the next release cycle. This procedure was repeated every 24 h for a total duration of 10 days. The resulting OD_600_ values reflected the antifungal activity of released CLT at each time point, providing a functional measure of drug release over time. As a control, identical procedures were carried out using drug-free networks (**IC-POSS** ^**a**^ **_COP** ^**b/c**^) that did not contain CLT. These control samples allowed for the evaluation of any intrinsic antifungal activity of the carrier systems and confirmed that the observed effects could be attributed to CLT release.

#### 2.10.3 Assessment of Antifungal Activity against Mature Biofilms

To obtain mature biofilms, 500 μL of a *C. albicans* suspension (either ATCC 10231 or SC5314) adjusted to 5 × 10^7^ CFU/mL in YPD medium buffered at pH 5.5 with 0.01 M citrate buffer was added to each well of a sterile 48-well microtiter plate and incubated at 37 °C for 24 h. After biofilm maturation, the supernatant containing planktonic cells was gently removed, and wells were washed twice with sterile phosphate-buffered saline (PBS) to remove nonadherent cells. The preformed biofilms were then treated with drug-free and drug-loaded networks. All samples, both with and without CLT, were sterilized by UV irradiation for 24 h in a laminar flow cabinet prior to use. Treatments containing 400 μg of CLT per well or equivalent amounts of unloaded copolymers, prepared in YPD medium buffered at pH 5.5 with 0.01 M citrate buffer, were applied to the biofilms. CLT (400 μg/well; 800 μg/mL) was used as a reference standard. Untreated biofilms served as negative controls. Following a 24 h incubation at 37 °C, biofilm viability was evaluated using the XTT [2,3-bis­(2-methoxy-4-nitro-5-sulfophenyl)-2H-tetrazolium-5-carboxanilide] reduction assay.^30^ Wells were washed twice with PBS to remove residual treatment, and then 500 μL of XTT solution (0.5 mg/mL XTT and 1 μM menadione in PBS) was added. Plates were incubated in the dark at 37 °C for 2 h. The metabolic activity of biofilm cells was quantified by measuring absorbance at 490 nm by using a BioTek SYNERGY H1 microplate reader (Agilent, Santa Clara, CA, USA). Experiments were performed in triplicate, and the biofilm viability was expressed as a percentage relative to untreated controls.

#### 2.10.4 Cytotoxicity Assessment Using the MTT Assay

The cytotoxicity of the tested materials was evaluated using the MTT (3-(4,5-dimethylthiazol-2,5-diphenyltetrazolium bromide)) (Sigma, St. Louis, MO, USA) assay^31^ on Human Normal Dermal Fibroblasts (NHDF; ATCC PCS-201-012). Cells were cultured in α-minimum essentials medium (α-MEM, Institute of Immunology and Experimental Therapy (IITD), Poland) supplemented with 1% glutamine (Sigma-Aldrich, St Louis, MO, USA), 1% antibiotics (10 U/mL penicillin and 10 μg/mL streptomycin, Sigma-Aldrich, St Louis, MO, USA) and 10% fetal bovine serum (FBS, Gibco, USA) and maintained at 37 °C in a humidified atmosphere with 5% CO_2_. For the assay, NHDF cells were seeded in 48-well plates at a density of 1 × 10^4^ cells per well and allowed to adhere for 24 h. After this preincubation period, the culture medium was replaced with fresh medium containing the tested drug-loaded networks (400 μg CLT/well), drug-free networks as controls, or CLT at a concentration of 400 μg/well (800 μg/mL). Wells containing untreated cells served as negative controls. Cells were exposed to the test compounds for 24 h. After incubation, cell viability was assessed by measuring the MTT formazan absorbance at 570 nm using a BioTek SYNERGY H1 microplate reader (Agilent, Santa Clara, CA, USA). The results were based on three independent experiments, each performed in triplicate and expressed as a percentage of the negative control.

#### 2.10.5 Statistical Analysis

All data are presented as the mean ± standard deviation (SD). Statistical significance was determined using Student’s *t*-test. The significance level was set at *P* < 0.05.

### 2.11 ^1^H NMR Spectroscopy in Solution

NMR measurements of open-POSS cages and their precursors were carried out using Bruker Avance III spectrometers operating at 500 MHz for ^1^H, ^13^C, and ^29^Si nuclei. Chemical shifts are reported in parts per million relative to tetramethylsilane, using the residual signals of the deuterated solvents as internal standards. Coupling constants (*J*) are expressed in hertz (Hz), and standard abbreviations—s (singlet), d (doublet), t (triplet), q (quartet), and m (multiplet)—are used to describe splitting patterns.

^1^H NMR spectra of DMAM-2-AAPBA copolymers were recorded using a Bruker Avance NEO AV 400 MHz. ^1^H DOSY measurements were carried out for both copolymers at 295 K on a Bruker Avance III 500 spectrometer equipped with a 5 mm BBI probe head with a z-gradient coil and a GAB/2 gradient unit capable of producing B0 gradients with a maximum strength of 50 G/cm. The BCU-05 cooling unit, managed by the BVT3300 variable temperature unit, was used for the temperature control and stabilization. The spectrometer was controlled with a PC running under Windows 7 (64 bit) OS with the TopSpin 3.1 program.

### 2.12 Solid-State ^11^B NMR Spectroscopy

The solid-state experiments were performed on a BRUKER Avance III 400 spectrometer operating at 128.38 MHz for ^11^B. For all measurements, the MAS probe head utilizing 4 mm ZrO_2_ rotors was used. Spectra were recorded with an MAS frequency of 8000 Hz.

For ^11^B spectra, the standard HPDec (high power decoupling) pulse program was utilized with ^11^B 90 deg. pulse of 4.0 μs in length and 9.0 μs 1 H 90 deg. pulse length in the spinal decoupling sequence. The repetition delay was 5 s, and the spectral width was 40.76 kHz. The FIDs were accumulated with a time domain size of 3584 data points, and 380 scans were accumulated for each FID. All spectra were collected and processed with the Top-Spin 2.1 software.

### 2.13 MALDI-MS Spectrometry

MALDI-MS spectra were recorded using a JEOL JMS-S3000 SpiralTOF-plus Ultra-High Mass Resolution MALDI-TOF/MS Mass Spectrometer.

### 2.14 Fourier Transform Infrared Spectroscopy, FTIR

FTIR spectra were obtained using a Shimadzu IRSpirit-T with a diamond ATR attachment.

### 2.15 Thermogravimetric and Differential Thermal Analysis (TG-DTA)

Thermogravimetric analysis was performed using a Setaram SETSYS 16/18 apparatus. Samples of the tested open POSS cages were placed in crucibles made of aluminum oxide (Al_2_O_3_). Measurements were conducted in an air atmosphere at a controlled flow rate of 1 dm^3^/h, over a temperature range of 30 to 1000 °C, with a linear heating rate of 10 °C/min.

### 2.16 Scanning Electron Microscopy–Energy-Dispersive X-ray Spectroscopy, SEM–EDS

SEM analyses were performed using a Hitachi S-3400N instrument equipped with a tungsten filament electron gun, offering magnification in the range of 80 to 300,000×. The instrument operated under low vacuum conditions (6–280 Pa), allowing for the examination of nonconductive samples without extensive preparation. Both secondary electron (SE) and backscattered electron (BSE) detectors were employed to obtain topographical and compositional contrast, respectively.

### 2.17 Elemental Analysis, EA

Elemental analysis was conducted using an integrated Thermo Scientific UltraDry EDS system with a lithium-drifted silicon (Si­(Li)) detector. When images were not sufficiently clear, samples were sputter-coated with a thin layer of gold by using a Cressington 108A coater to enhance surface conductivity.

### 2.18 Differential Scanning Calorimetry, DSC

The thermal properties of the neat DMAM-2-AAPBA copolymers, CLT, drug-free, and drug-loaded polymer networks were evaluated using a DSC [TA Instruments (2500 Discovery series)]. A specific amount of sample was heated in a sealed aluminum pan from room temperature to 160 °C and then cooled to −80 °C and heated to 160 °C again with a heating/cooling rate of 10 °C/min.

### 2.19 Gel Permeation Chromatography, GPC

The average molecular weight of DMAM-2-AAPBA copolymers was determined with gel permeation chromatography (GPC) using a Shimadzu Pump LC-20AD instrument and a Shimadzu SIL-20A HT Autosampler. An RI-Optilab T-rex-Wyatt refractometer and a DAWN 8+ laser photometer (Wyatt Technology) were used as detectors. *N*,*N*′-Dimethylformamide was used as the eluent at a flow rate of 0.8 mL/min at 25 °C.

### 2.20 Rheology

The oscillation frequency sweep tests were performed on a Thermoscientific HAAKE MARS 40 rheometer in the linear viscoelastic regime using a parallel plate–plate geometry of 8 mm diameter with a 0.5 mm gap at 37 °C.

## 3 Results and Discussion

### 3.1 Synthesis of Diol-Functionalized **IC-POSS** ^**R**^ (R = iBu, Ph) Components

The synthesis of **IC-POSS** ^**R**^ (R = iBu, Ph) species was carried out via a two-step procedure (Scheme 1). Initially, commercially available trisilanol-type open-cage silsesquioxanes, i.e., trisilanolisobutyl-POSS and trisilanolphenyl-POSS, were subjected to a reaction with dimethylchlorosilane (Scheme 1, step 1), yielding hepta­(isobutyl)­tris­(dimethylsiloxy)-POSS and hepta­(phenyl)­tris­(dimethylsiloxy)-POSS, respectively. This first stage aimed to extend the silanol arm to increase the likelihood of substitution and the formation of a dimethylsiloxy linker; the direct substitution of silanol moieties is hindered by the presence of isobutyl or phenyl substituents anchored to the neighboring silicon atoms of the inorganic core. The resulting intermediates were thoroughly analyzed using multinuclear NMR spectroscopy (^1^H, ^13^C, and ^29^Si), FT-IR spectroscopy, and MALDI-TOF mass spectrometry.

**Scheme 1:**
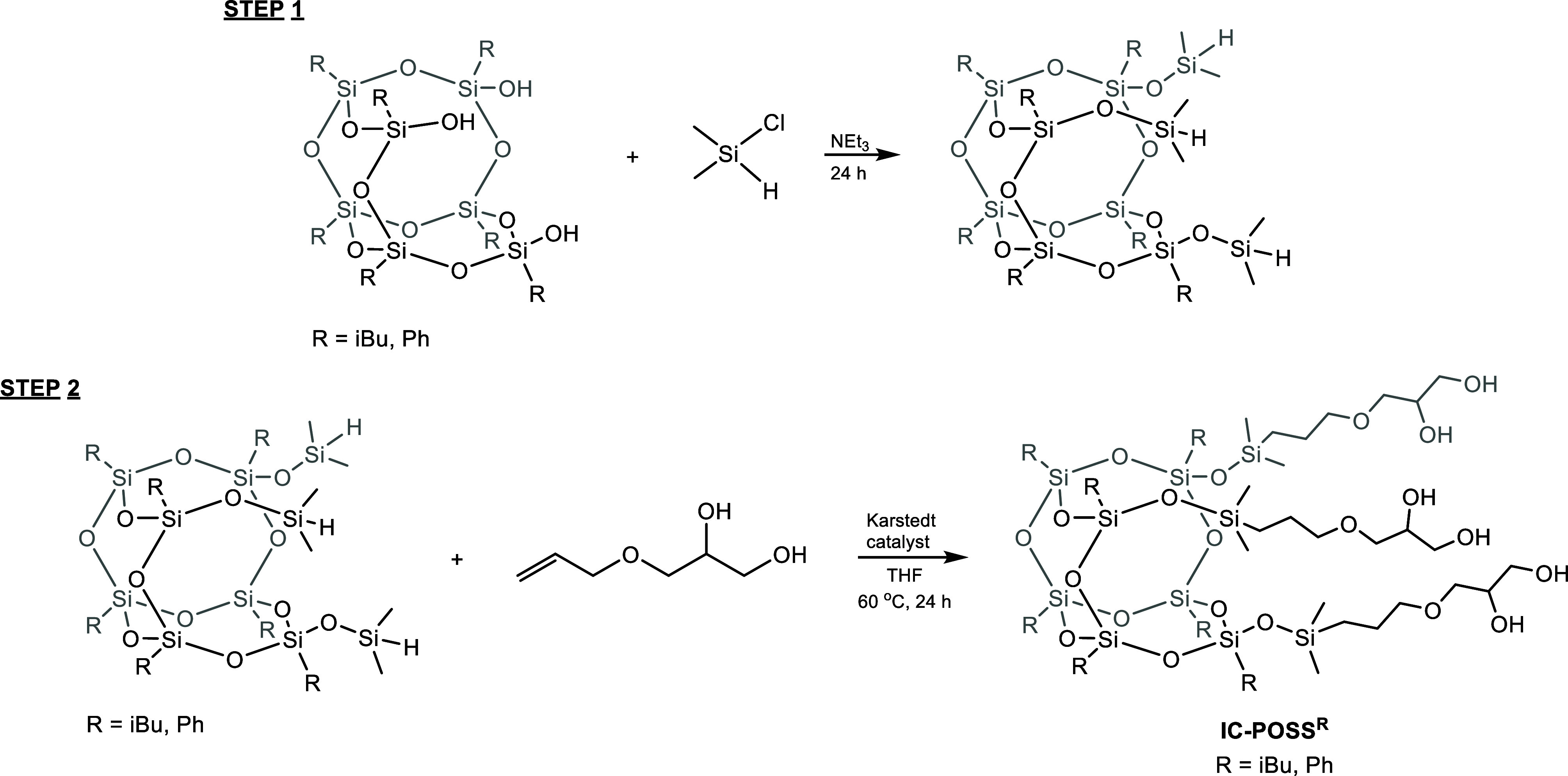
Two-Step Synthesis of **IC-POSS** ^**R**^ (R = iBu and Ph) Components

In the ^1^H NMR spectrum (Figure S1) of hepta­(isobutyl)­tris­(dimethylsiloxy)-POSS, the characteristic septet at 4.75 ppm corresponds to the silyl protons from the siloxy linker, while the septet observed between 1.88 and 1.82 ppm arises from the methine protons adjacent to the methyl and methylene centers. The large doublet at 0.96 ppm is attributable to the terminal methyl groups of the isobutyl substituents; the smaller doublet at 0.57 ppm corresponds to the methylene group of the isobutyl substituents. The signal at 0.23 ppm is assigned to the methyl protons of the dimethylsiloxy moieties. The ^13^C NMR spectrum (Figure S2) shows resonances at 25.32, 25.20, 23.99, and 23.43 ppm for the various aliphatic carbons of the isobutyl arms, with the methyl carbons from the siloxy linker appearing at 0.00 ppm. In the ^29^Si NMR spectrum (Figure S3), the silicon environment of the T_8_ core is observed at −67.12 (1Si), −67.69 (3Si), and −68.02 (3Si) ppm, whereas the tris­(dimethylsiloxy) substituents give a distinct resonance at −5.49 (3Si) ppm. IR spectroscopy confirms the presence of key functional groups: weak C–H stretches at 2953 and 2869 cm^–1^, a Si–H band at 2140 cm^–1^, and a very strong Si–O–Si asymmetric stretch at 1052 cm^–1^, alongside weaker Si–C and Si–O–Si bending modes at 762 and 473 cm^–1^, respectively (Figure S4). Finally, high-resolution MALDI-MS displays a sodium adduct peak at *m*/*z* 987.387, in excellent agreement with the calculated [M + Na]^+^ of 987.36 (Figure S5).

Similarly, the structural assignment of hepta­(phenyl)­tris­(dimethylsiloxy)-POSS was established through complementary spectroscopic techniques. In the ^1^H NMR spectrum (Figure S7), the aromatic region features a doublet at 7.60 ppm, overlapping multiplets from 7.50 to 7.39 ppm, 7.35 to 7.24 ppm, and 7.19 to 7.12 ppm, consistent with the seven phenyl substituents. A distinctive septet at 4.94 ppm corresponds to the silyl protons from the siloxy linker, while the methyl groups give rise to a doublet at 0.36 ppm. The ^13^C NMR spectrum (Figure S8) corroborates these assignments, showing signals at 133.35, 131.79, 129.42, and 126.85 ppm for the aromatic carbons and a resonance at 0.00 ppm for the methyl carbons from the siloxy group. In the ^29^Si NMR spectrum (Figure S9), the T_8_ cage silicon environments appear at −77.27 (1Si), −77.61 (3Si), and −78.24 (3Si) ppm, whereas the three siloxy-attached silicon atoms resonate at −10.25 (3Si) ppm. Infrared analysis reveals weak aromatic C–H stretches at 3075 cm^–1^, aliphatic C–H stretches at 2963 cm^–1^, a Si–H band at 2132 cm^–1^, and a very strong Si–O–Si asymmetric stretch at 1053 cm^–1^, along with characteristic bending modes at 895, 694, 578, and 483 cm^–1^ (Figure S10). High-resolution MALDI-MS shows a sodium-adduct peak at *m*/*z* 1127.133, in close agreement with the calculated [M + Na]^+^ value of 1128.8 (Figure S11). In step 2 (Scheme 1), the resulting intermediates were reacted with 3-allyloxy-1,2-propanediol to yield diol-functionalized **IC-POSS** ^**iBu**^ and **IC-POSS** ^**Ph**^. Products were characterized by NMR (^1^H, ^13^C, ^29^Si), FT-IR, and MALDI-TOF MS. For compound **IC-POSS** ^**iBu**^ in the ^1^H NMR spectrum (Figure S13), multiplets at 3.84, 3.67, 3.60, 3.47, and 1.58 ppm and a singlet at 3.41 ppm are assigned to the methylene and methine protons of the 3-allyloxy-1,2-propanediol moiety and adjacent siloxy linkers. A singlet at 2.69 ppm corresponds to hydroxyl groups. The broad multiplet at 1.82 ppm and the additional multiplet at 0.53 ppm arise from the isobutyl CH and CH_2_ protons, respectively. The doublet of doublets at 0.94 ppm reflects the methyl groups of the seven isobutyl substituents. Further upfield resonances at 0.61 ppm are attributed to methylene groups attached to silicon, and a singlet at 0.12 ppm confirms the presence of methyl groups from dimethylsiloxy units. The ^13^C NMR spectrum (Figure S14) exhibits signals at 72.19, 71.54, 70.27, and 63.87 ppm for the diol and allyloxy carbons, resonances at 25.75 and 25.58 ppm for isobutyl methyl and methylene carbons, and 23.81 and 23.71 ppm for methylene carbons, 13.71 ppm for isobutyl methine, and 0.00 ppm for dimethylsiloxy methyl carbons. In the ^29^Si NMR spectrum (Figure S15), four singlets at 9.15 (3Si), −67.25 (1Si), −67.59 (3Si), and −67.83 (3Si) ppm confirm multiple silicon environments characteristic of an incompletely condensed T_8_ framework. The IR spectrum (Figure S16) displays a weak O–H stretch at 3347 cm^–1^, C–H stretches at 2962 and 2875 cm^–1^, a C–O stretch at 1259 cm^–1^, and a very strong Si–O–Si band at 1052 cm^–1^, with additional deformations at 696 and 483 cm^–1^. MALDI-TOF mass spectrometry shows a sodium adduct at *m*/*z* 1383.62, in excellent agreement with the calculated [M + Na]^+^ mass of 1383.59, thereby confirming the proposed composition and degree of functionalization (Figure S17).

In turn, in the ^1^H NMR spectrum of **IC-POSS** ^**Ph**^ (Figure S19) there are three distinct aromatic regions at 7.38, 7.33–7.28, and 7.12 ppm, consistent with aromatic protons, alongside signals at 3.81, 3.66, 3.57 ppm and multiplets at 1.68–1.60 ppm and 3.48–3.35 ppm attributable to the diol and allyloxy methylene and methine protons. A signal at 2.81 ppm corresponds to hydroxyl groups. A singlet at 1.83 ppm likely arises from residual isobuthyl methine fragments. While a multiplet at 0.71–0.56 ppm reflects methylene protons attached to the silicon in the reactive group. A sharp singlet at 0.25 ppm corresponds to six equivalent dimethylsiloxy methyl groups. The ^13^C NMR spectrum (Figure S20) shows aromatic carbons at 133.60, 132.37, 130.75, and 127.32 ppm, diol/allyloxy carbons at 74.01, 71.98, 70.41, and 63.81 ppm, alkyl carbons at 22.95 and 13.68 ppm, and the dimethylsiloxy methyl carbons at 0.00 ppm. In the ^29^Si NMR (Figure S21), four singlets at 11.93 (3Si), −77.35 (1Si), −77.55 (3Si) and −77.97 (3Si) confirm multiple silicon environments characteristic of an incompletely condensed T_8_ framework. Infrared spectroscopy displays a weak O–H stretch at 3375 cm^–1^, C–H stretches at 2956 and 2870 cm^–1^, aromatic C=C stretch at 1593 cm^–1^, C–O stretch at 1259 cm^–1^, and a very strong Si–O stretch at 1047 cm^–1^ with additional Si–O–Si and Si–C deformations at 483 and 696 cm^–1^, respectively (Figure S22). Finally, MALDI-TOF mass spectrometry shows a [M + Na]^+^ peak at *m*/*z* 1523.38, in excellent agreement with the calculated mass of 1523.37, fully corroborating its structure (Figure S23).

All open-POSS derivatives show high thermal stability, with 10% mass loss (Δ*T*_10%_) at 291.4–422.2 °C. Phenyl-substituted compounds decompose at higher temperatures due to aromatic resonance and steric effects (Figures S6, S12, S18, S24).

### 3.2 Formation of Cross-Linked Polymeric Films

A series of drug-free and drug-loaded films were fabricated via the cross-linking of open-POSS cages, specifically those bearing phenyl (**IC-POSS** ^**Ph**^) or isobutyl (**IC-POSSi** ^**Bu**^) functionalities, with poly­(dimethylacrylamide-2-acrylamidephenylboronic acid) (P­(DMAM-2-AAPBA)) through boronic ester linkages (Scheme 2). Film preparation consistently maintained an equimolar ratio of 1,2-diol to boronic acid groups, employing two distinct P­(DMAM-2-AAPBA) copolymers characterized by DMAM-2-AAPBA molar ratios of 8:2 (**COP** ^**8/2**^) and 9:1 (**COP** ^**9/1**^),Table 2.

**Scheme 2:**
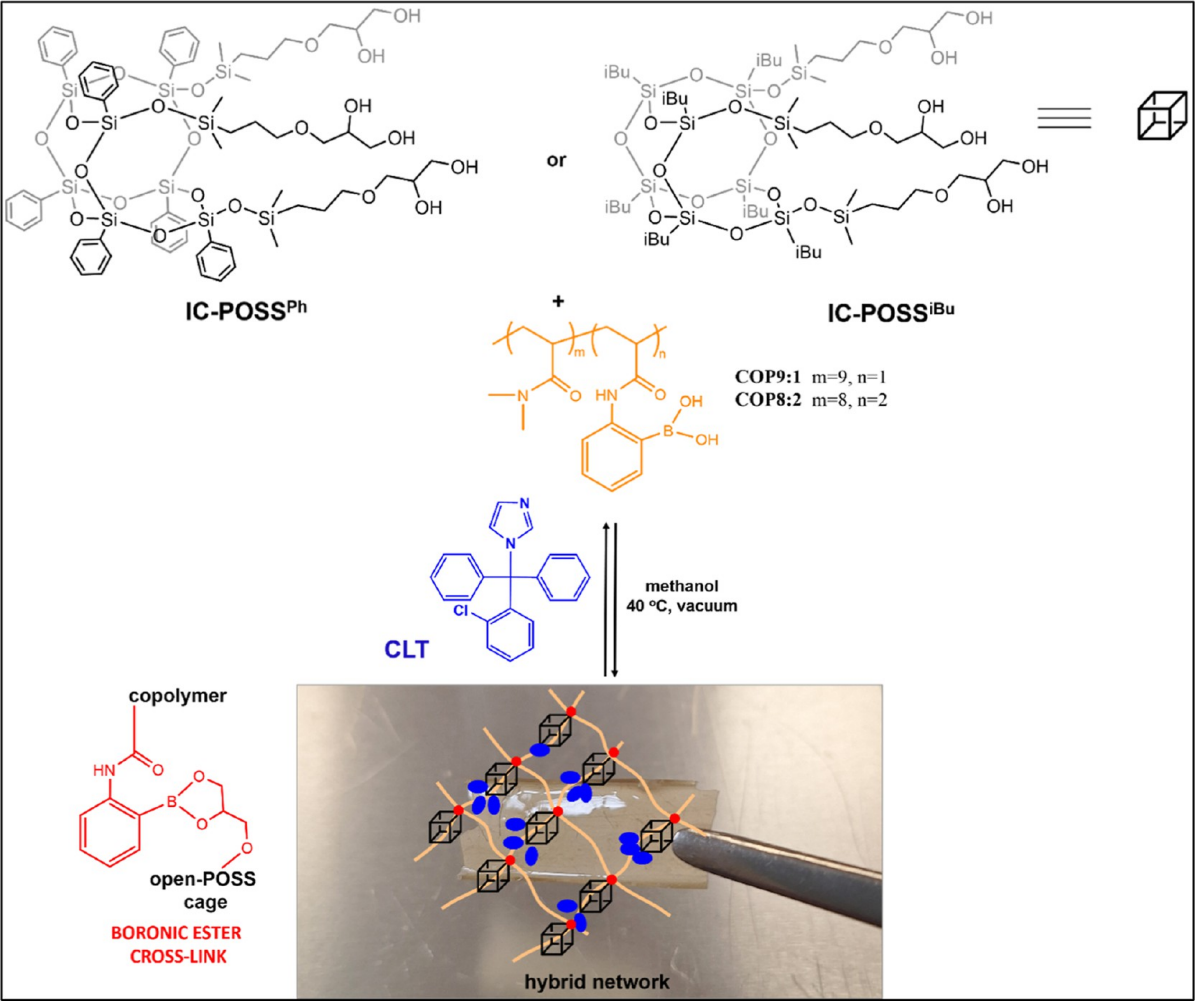
Schematic Representation of Clotrimazole-Loaded Films Construction Based on the Reversibly Cross-Linking Open-POSS Cages (**IC-POSS** ^**Ph**^ or **IC-POSS** ^**iBu**^) with Dimethylacrylamide-2-acrylamidephenylboronic Acid Copolymers Differing in the Molar Ratio of Comonomers (**COP** ^**9/1**^ or **COP** ^**8/2**^)

**Table 2:** Composition of Films Constructed of Cross-Linked **IC-POSS** ^**Ph**^ and **IC-POSS** ^**iBu**^ Cages, Respectively, with DMAM-2-AAPBA Copolymers (**COP** ^**9/1**^ or **COP** ^**8/2**^, Respectively)

film	cage	copolymer	molar ratio of clotrimazole to cage	theoretical molar ratio of 1,2-diol to 2-AAPBA	weight fraction of clotrimazole in the network, wt %
**IC-POSS** ^**Ph**^ **_COP** ^**9/1**^ **_CLT** ^**2**^	Ph	**COP** ^**9/1**^	2	1	12.7
**IC-POSS** ^**Ph**^ **_COP** ^**8/2**^ **_CTL** ^**2**^	Ph	**COP** ^**8/2**^	2	1	17.5
**IC-POSS** ^**Ph**^ **_COP** ^**9/1**^ **_CTL** ^**4**^	Ph	**COP** ^**9/1**^	4	1	22.5
**IC-POSS** ^**Ph**^ **_COP** ^**8/2**^ **_CTL** ^**4**^	Ph	**COP** ^**8/2**^	4	1	29.7
**IC-POSS** ^**iBu**^ **_COP** ^**9/1**^ **_CLT** ^**2**^	iBu	**COP** ^**9/1**^	2	1	13.0
**IC-POSS** ^**iBu**^ **_COP** ^**8/2**^ **_CLT** ^**2**^	iBu	**COP** ^**8/2**^	2	1	18.1
**IC-POSS** ^**iBu**^ **_COP** ^**9/1**^ **_CLT** ^**4**^	iBu	**COP** ^**9/1**^	4	1	23.0
**IC-POSS** ^**iBu**^ **_COP** ^**8/2**^ **_CLT** ^**4**^	iBu	**COP** ^**8/2**^	4	1	30.6
**IC-POSS** ^**Ph**^ **_COP** ^**9/1**^	Ph	**COP** ^**9/1**^		1	
**IC-POSS** ^**Ph**^ **_COP** ^**8/2**^	Ph	**COP** ^**8/2**^		1	
**IC-POSS** ^**iBu**^ **_COP** ^**9/1**^	iBu	**COP** ^**9/1**^		1	
**IC-POSS** ^**iBu**^ **_COP** ^**8/2**^	iBu	**COP** ^**8/2**^		1	

The preparation of drug-free networks involved dissolving both the open-POSS cage and the P­(DMAM-2-AAPBA) copolymer in methanol. For drug-loaded films, clotrimazole was codissolved with these building components in methanol. Clotrimazole was incorporated into the networks at molar ratios of 2:1 and 4:1 per open-POSS cage. Network formation proceeded through solvent evaporation with an initial ambient pressure evaporation step followed by vacuum drying at 45 °C for complete methanol removal.

We explored other 2-AAPBA copolymers with such comonomers as *N*-isopropylacrylamide or acrylamide for cross-linking the open-POSS cages. However, achieving homogeneous drug-loaded networks with these alternatives has proved challenging. The superior capability of DMAM-2-AAPBA copolymers in achieving the desired uniform drug dispersion in the networks makes them the ideal component for our film formulations.

### 3.3 Characteristics of Cross-Linked Polymeric Films

#### 3.3.1 Solid-State ^11^B NMR Spectroscopy

Solid-state ^11^B NMR spectroscopy was used to confirm network formation via boronic ester cross-links in both drug-free and drug-loaded films. For the identification of both 2-acrylamidephenylboronic acid and its ester in the networks, ^11^B NMR spectra were initially recorded in the solid state for low-molecular-weight compounds, i.e., both pinacol ester of 2-acrylamidephenyl boronic acid, 2-acetamidophenyl boronic acid, and copolymers varying molar ratios of dimethylacrylamide and 2-acrylamidephenylboronic acid, i.e., 9:1 and 8:2, for **COP** ^**9/1**^ and **COP** ^**8/2**^ (Figure 1), respectively. The signal of the boron atom in 2-acetamidophenyl boronic acid at −2.9 ppm was shifted to 0.3 ppm for its ester counterpart, i.e., pinacol ester of 2-acrylamidephenyl boronic acid. The signal of boronic acid in the 2-AAPBA constitutional units in both copolymers was located at −1.2 ppm. ^11^B NMR spectra recorded for neat and drug-loaded networks revealed the formation of boronic ester cross-links between 1,2-diol groups of the open-POSS cage and boronic acid of the DMAM-2-AAPBA copolymer, as evidenced by a signal at 2.9 ppm. In spite of using an equimolar diol and boronic acid mixture, the population of boronic acid in each investigated network was still present, which indicated that a certain molar fraction of 1,2-diol groups of each of the investigated cages remained intact.

**Figure 1:**
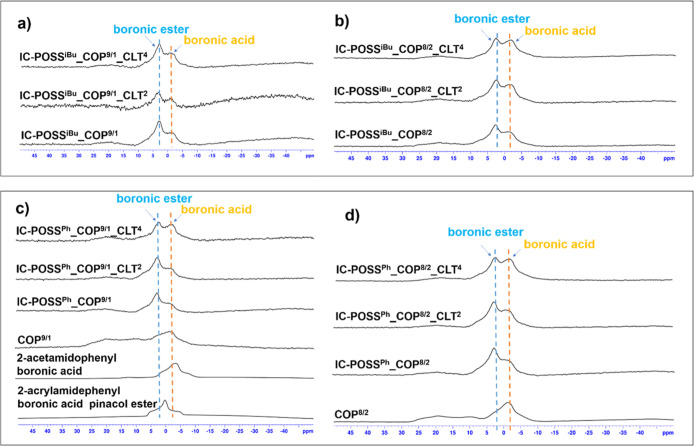
Comparison of ^11^B NMR spectra recorded for neat networks, drug-loaded networks constructed of **IC-POSS** ^**iBu**^ and **IC-POSS** ^**Ph**^ cages, respectively, cross-linked via **COP** ^**9/1**^ (a,c) and **COP** ^**8/2**^ (b,d) with low molecular boronic acid, boronic ester, and **COP** ^**9/1**^ (c) and **COP** ^**8/2**^(d).

Moreover, we noticed that the molar fraction of formed boronic esters is strictly dependent on the amount of incorporated drug. For all systems based on the cross-linked **IC-POSS** ^**Ph**^ or **IC-POSS** ^**iBu**^, except **IC-POSS** ^**iBu**^ **_COP** ^**9/1**^ **_CLT** ^**4**^ sample, the higher the molar fraction of drug that was used, the lower the fraction of formed boronic ester species that was observed. This behavior can be a result of the phase separation of the drug and polymer network, as visible in SEM-EDS images (Table 3). The effect was stronger with the phenyl-rich cage, likely due to π–π interactions with clotrimazole causing steric hindrance and limiting access to reactive groups.

**Table 3:**
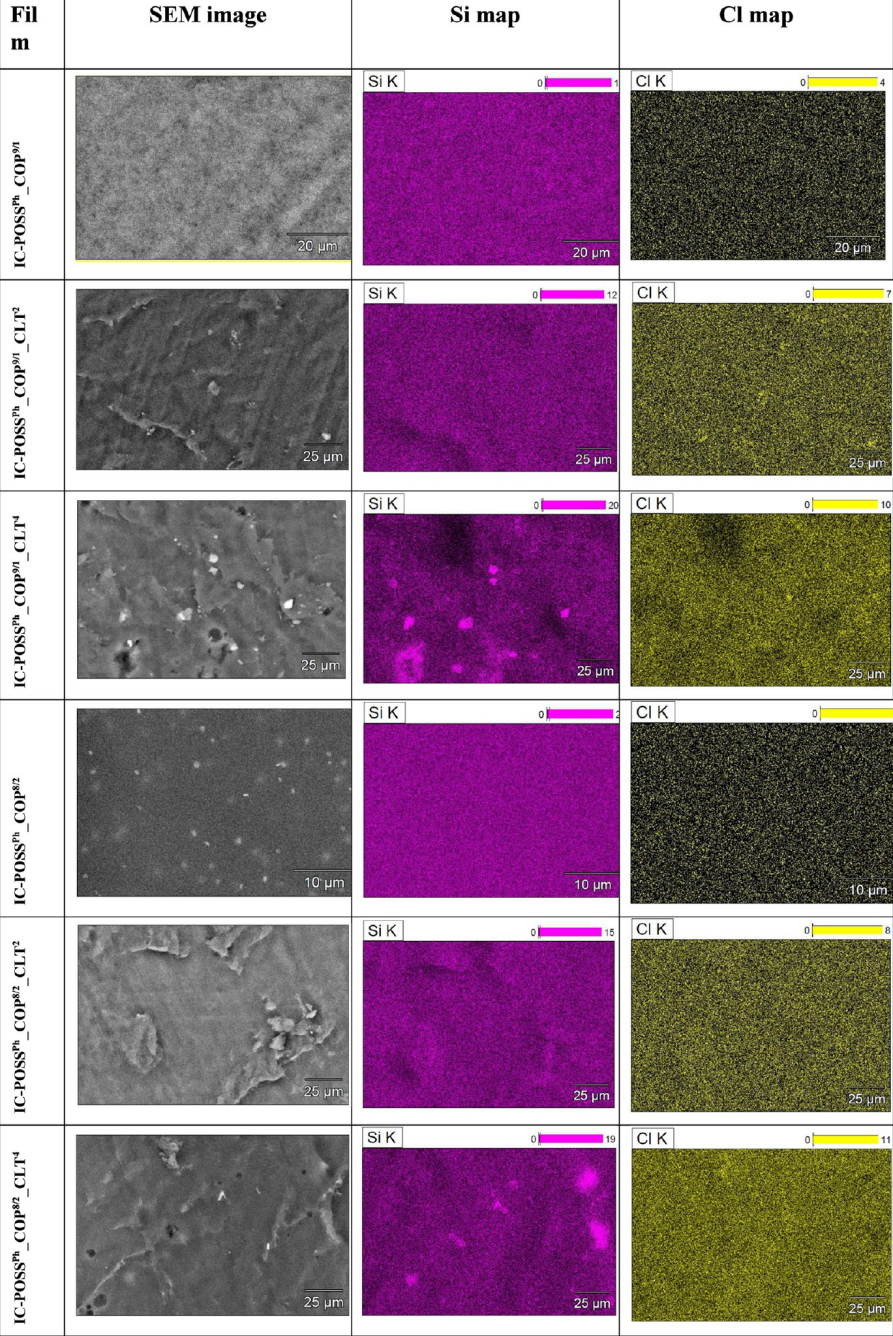
Comparison of SEM–EDS Images Recorded for Neat- and Drug-Loaded Films Constructed of **IC-POSS** ^**Ph**^ Cross-Linked via DMAM-2-AAPBA Copolymers

#### 3.3.2 DSC Analysis

Detailed DSC analysis performed for neat DMAM-2-AAPBA copolymers and their networks based on cross-linked open-POSS cages via boronic esters revealed the differences in the segmental mobility of polymer chains caused by the cross-linking. For the study of segmental mobility of polymer chains, thermograms of the second heating were taken into consideration. DSC analysis of DMAM-2-AAPBA copolymers showed that neat **COP** ^**8/2**^ displays a glass transition (*T*_g_ = 93.2 °C) lower than that of **COP** ^**9/1**^ (*T*_g_ = 99.1 °C), which is a result of a higher molar fraction of 2-AAPBA units in the copolymer. The covalent cross-linking of 2-AAPBA units of both copolymers with 1,2-diol groups of open-POSS cages resulted in the decrease of *T*_g_ (Figure 2), which is associated with the increase of the “free volume” between polymer chains, enabling more segmental movement of the polymer chains, making the material softer.^32^ A lower reduction in the *T*_g_ value was, however, observed for networks constructed of **COP** ^**8/2**^, which can be explained by a more dense network formed and, consequently, greater restriction of polymer segment mobility. For instance, networks constructed of **COP** ^**8/2**^ and an open-POSS cage bearing phenyl moieties (**IC-POSS** ^**Ph**^), exhibited a decrease in glass transition temperature *T*_g_ to 82.3 °C (Figure 2). In comparison, networks based on **COP** ^**9/1**^ showed a decrease in *T*_g_ to 69.3 °C. Similarly, for films made from cross-linked isobutyl-enriched open-POSS cages (**IC-POSS** ^**iBu**^), a reduction in *T*_g_ to 87.6 and 56.7 °C was observed for **COP** ^**8/2**^ and **COP** ^**9/1**^, respectively.

**Figure 2:**
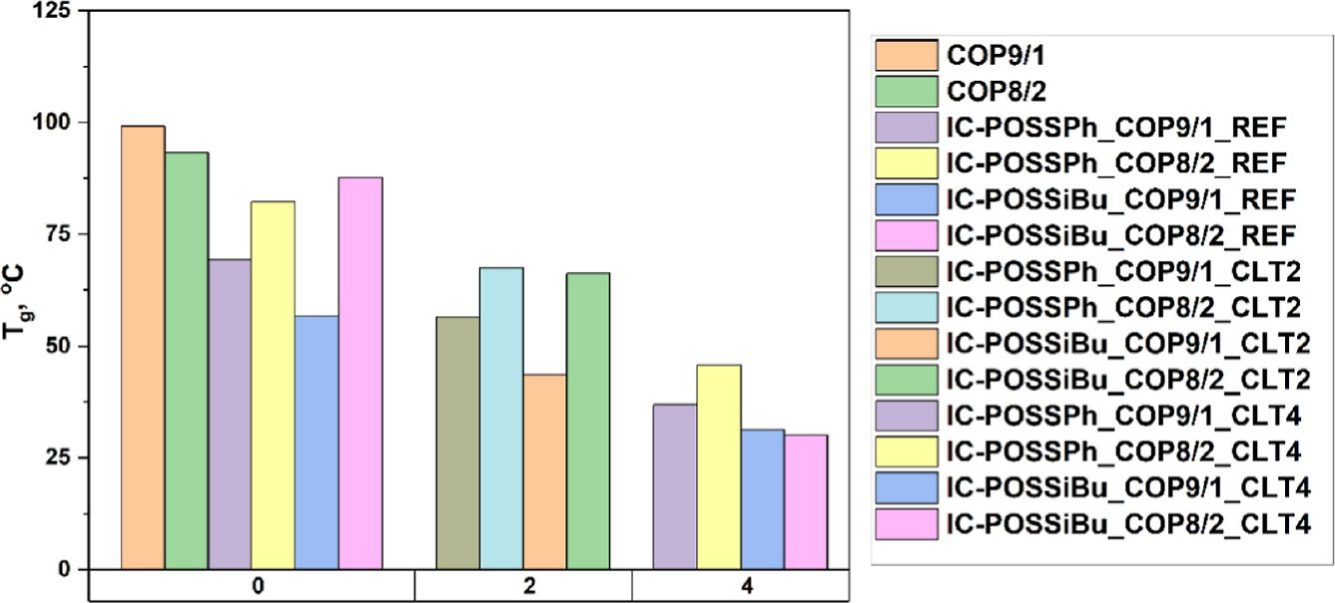
Comparison of glass transition recorded for neat copolymers, drug-free, and drug-loaded networks constructed of copolymer cross-linked with open-POSS cages.

It is noteworthy that the polymer mobility in the synthesized drug-free networks was strictly dependent on the type of cage used. *T*_g_ of networks constructed of **IC-POSS** ^**Ph**^ were stiffer than systems based on **IC-POSS** ^**iBu**^, which can be an effect of intermolecular π–π interactions taking place between phenyl moieties of individual cages in the network. This behavior was also visible for drug-loaded networks at a molar ratio of drug molecules to one cage equal to 2:1.

DSC showed that increasing the clotrimazole content lowered *T*_g_ for polymer chains in networks with both **IC-POSS** ^**Ph**^ and **IC-POSS** ^**iBu**^ cages (Figure 2). For instance, the incorporation of drug molecules into the network composed of the cross-linked **IC-POSS** ^**Ph**^ **_COP** ^**9/1**^ resulted in the decrease of *T*_g_ from 69.3 °C to 56.5 and 36.9 °C for the networks in which 2 and 4 drug molecules were used per one cage, i.e., **IC-POSS** ^**Ph**^ **_COP** ^**9/1**^ **_CLT** ^**2**^ and **IC-POSS** ^**Ph**^ **_COP** ^**9/1**^ **_CLT** ^**4**^, respectively. This behavior shows that clotrimazole molecules play the role of a plasticizer; i.e., they enhance the mobility of polymer segments in the network, causing its softening.

DSC thermograms (Figure 3, first heating) showed that cross-linking during film formation prevents drug crystallization, regardless of the copolymer or open-POSS cage used. This was evidenced by the lack of endothermic peak at 145.5 °C for the obtained networks, which is typical for the melting of pure drug crystals. The absence of this signal indicates that the structure of constructed films prevents the drug crystallization, making the drug more bioavailable. DSC of solvent-free clotrimazole–POSS mixtures showed that only **IC-POSS** ^**Ph**^ prevented drug crystallization, suggesting strong compatibility likely due to π–π interactions with clotrimazole. Such interactions are crucial for ensuring a homogeneous drug distribution within the network. Furthermore, the absence of clotrimazole’s melting point in films constructed with cross-linked **IC-POSS** ^**iBu**^ cages definitively demonstrates that DMAM-2-AAPBA copolymers play an additional critical role in achieving homogeneous drug distribution within the network.

**Figure 3:**
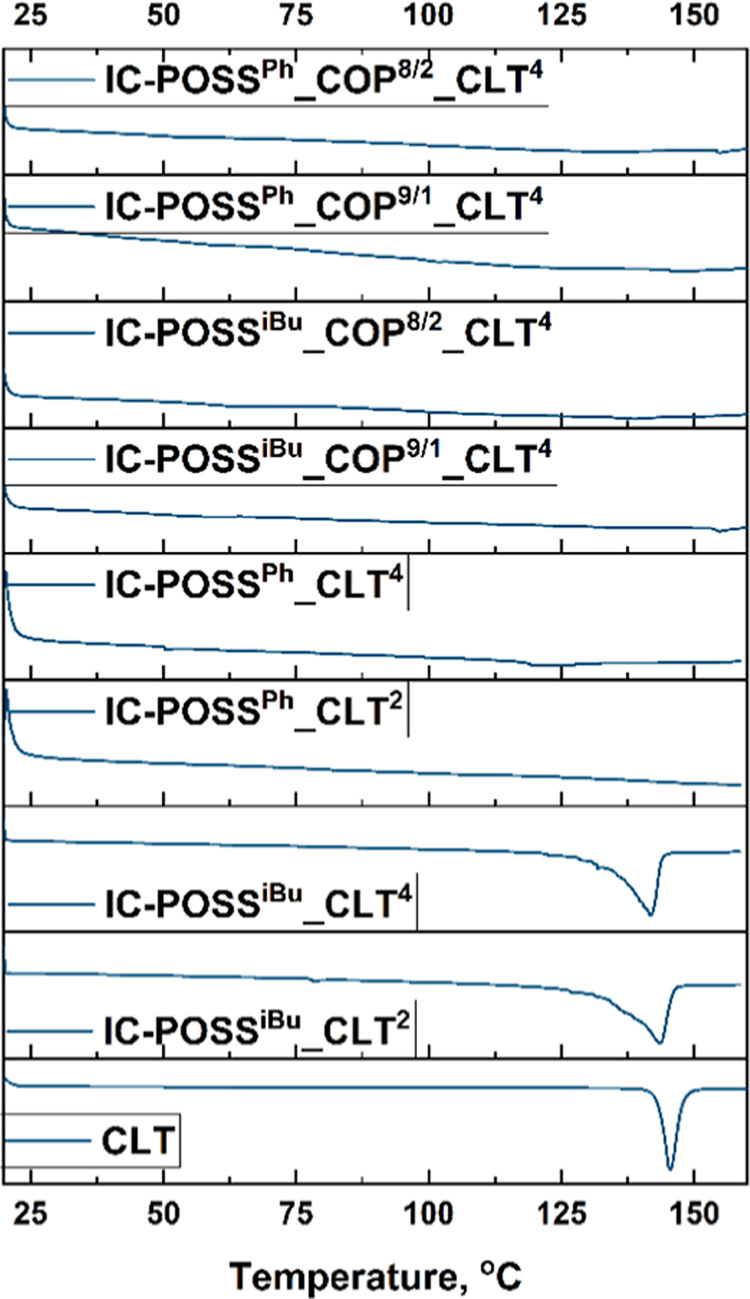
Comparison of DSC thermograms obtained in the first heating for clotrimazole, its mixture with open-POSS cages, and clotrimazole-loaded networks constructed of DMAM-2-AAPBA copolymers cross-linked with open-POSS cages.

#### 3.3.3 SEM–EDS Analysis

SEM–EDS analysis was performed on both neat and drug-loaded networks to demonstrate the distribution of both kinds of building components within the material, i.e., open-POSS cage and DMAM-2-AAPBA copolymers on the one hand and clotrimazole on the other. The distribution of IC-POSS cages within the networks was monitored based on the identification of silica in the composition map, whereas clotrimazole distribution was analyzed based on the presence of a chlorine atom.

SEM–EDS revealed that both **IC-POSS** ^**Ph**^ and **IC-POSS** ^**iBu**^ cages are compatible with both types of used DMAM-2-AAPBA copolymers, i.e., **COP** ^**9/1**^, and **COP** ^**8/2**^, and form homogeneous formulations (Tables 3 and 4). Incorporation of clotrimazole at a 2:1 molar ratio (drug to cage) into film formulations based on **IC-POSS** ^**Ph**^ cages cross-linked with a DMAM-2-AAPBA (9:1) copolymer resulted in homogeneous networks. Increasing the molar ratio of clotrimazole to the cage to 4:1 in the network resulted in the formation of a few aggregates composed mainly of silsesquioxane cages (Table 3), which indicates that a higher amount of clotrimazole in the network can stimulate the aggregation of silsesquioxane cages. The formation of **IC-POSS** ^**Ph**^-based aggregates may reduce the availability of 1,2-diol groups, which may explain the decrease in the molar fraction of boron ester cross-links present in this formulation, as seen in the ^11^B NMR spectrum. However, the use of **COP** ^**8/2**^ for the **IC-POSS** ^**Ph**^ cage cross-linking allowed for the production of homogeneous formulations regardless of the molar ratio of the drug to the cage used. The incorporation of drug into the formulations based on **IC-POSS** ^**iBu**^ cross-linked with DMAM-2-AAPBA, 9:1 copolymer in the molar ratio of drug to the cage, both 2:1 and 4:1, respectively, resulted in films with visibly separated drug in the form of objects below 25 μm. Using **COP** ^**8/2**^, i.e., a copolymer with a higher 2-AAPBA content, for **IC-POSS** ^**iBu**^ networks formation prevented drug precipitation, likely due to increased network density and better drug dispersion. In summary, SEM-EDS analysis also confirmed the DSC data, indicating that **IC-POSS** ^**Ph**^ cages are more compatible with clotrimazole molecules than **IC-POSS** ^**iBu**^. However, the use of copolymer characterizing higher molar content of 2-AAPBA units (**COP** ^**8/2**^) for cross-linking of **IC-POSS** ^**iBu**^ cages improves the drug distribution in the network.

**Table 4:**
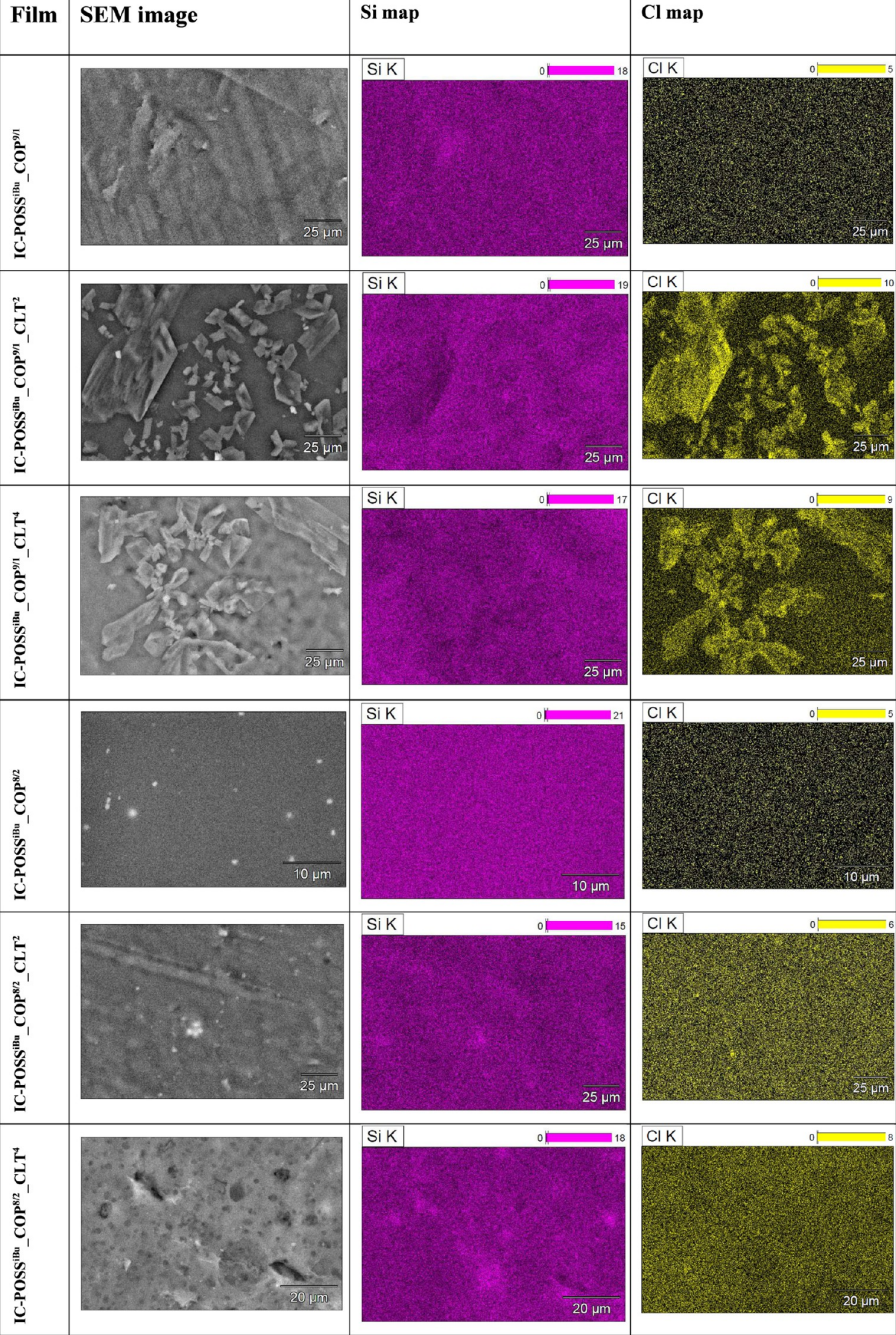
Comparison of SEM–EDS Images Recorded for Neat- and Drug-Loaded Films Constructed of **IC-POSS** ^**iBu**^ Cross-Linked via DMAM-2-AAPBA Copolymers

It is worth noting that DSC analysis performed on film preparations containing the drug after half-year storage at room temperature showed no differences in either the first or second heating cycle. No formation of the crystalline form of the drug, which is prevented by the network, was observed.

#### 3.3.4 Characteristics of Water-Swollen Drug-Loaded Network-Based Films

##### 3.3.4.1 Swelling Behavior of Films and Their Rheological Characteristics

The water-swelling behavior of drug-loaded films is a crucial parameter for their successful intravaginal application. On one hand, controlled swelling facilitates adhesion, ensuring prolonged retention of the drug carrier at the application site. On the other hand, swelling properties directly influence the kinetics of drug release and maintain the structural integrity of the film within the physiological environment. Samples of dry drug-loaded films were able to swell with water; however, the swelling ratio of the drug carrier was strictly dependent on the network composition, i.e., the kind of cage used, copolymer, and amount of drug incorporated into the networks (Figure 4a). The samples were immersed in deionized water for 15 min. The swelling ratio of each network was estimated according to the following equation:^33^ $S=\frac{\left(w_{w}-w_{d}\right)\;\cdot \;100\%}{w_{d}}$ , where *w*_w_—mass of the swollen wet hydrogel sample, *w*_d_—mass of the dried sample. The highest swelling ratio was observed for films which were constructed of **COP** ^**9/1**^, but this effect was mainly evident in networks based on the cross-linked **IC-POSS** ^**iBu**^ cages, which characterized lower *T*_g_ referring to increased free volume between chains, causing more effective network penetration with water molecules. Furthermore, the higher the molar fraction of the drug used per cage in a given network type, the higher the swelling ratio observed. It was strictly associated with higher segmental motion of polymer chains^34^ in the network.

**Figure 4:**
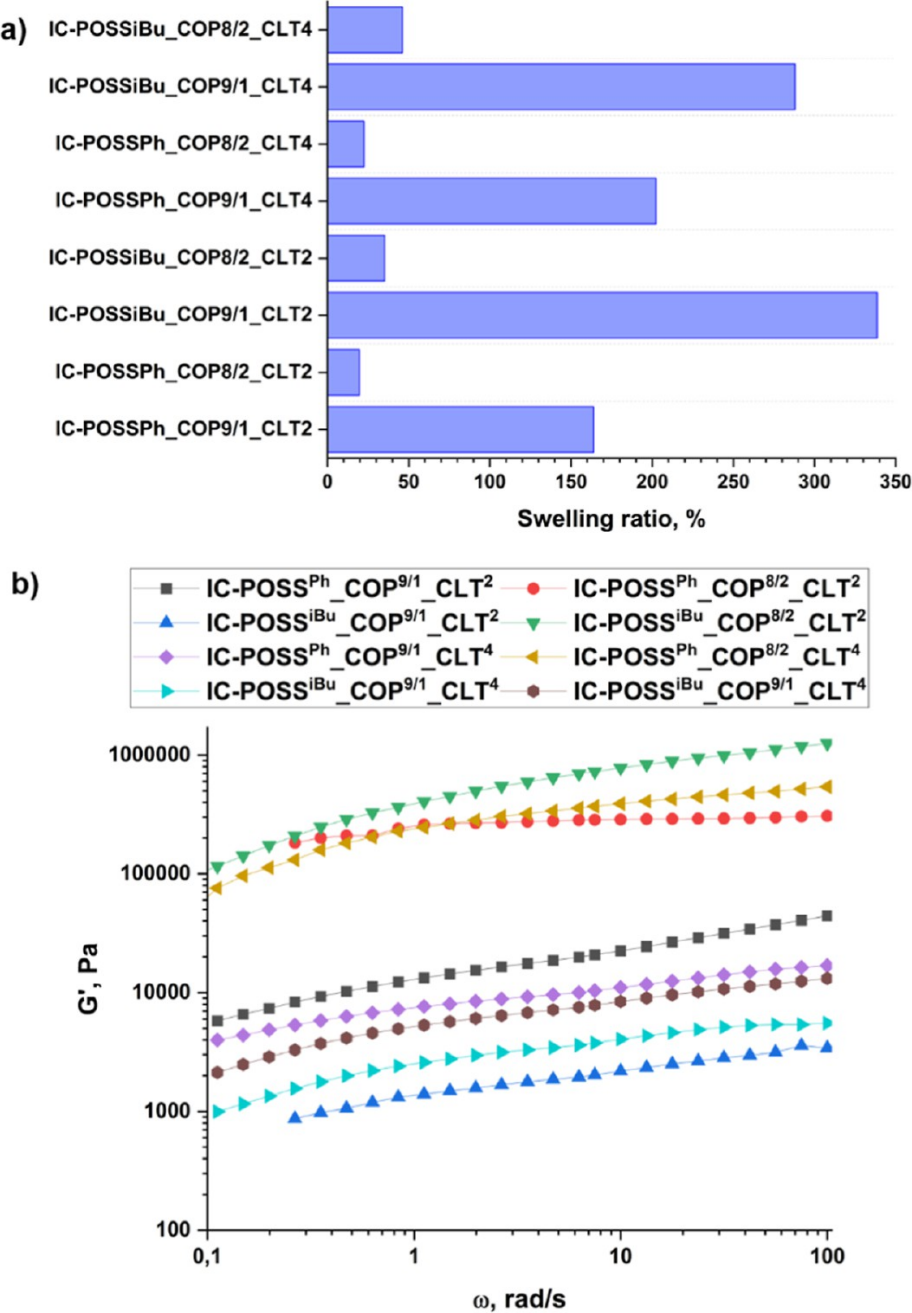
Swelling behavior of drug-loaded films incubated in water for 15 min (a). Comparison of storage modulus recorded for hydrated drug-loaded films (b).

Rheological investigations, conducted via frequency sweeps (0.1 to 100 rad/s) on drug-loaded films swollen in water for 15 min, demonstrated that the storage modulus (G′) consistently surpassed the loss modulus (G″). These data revealed a dominant solid-like behavior for the synthesized systems (Figures S29 and S30). Further analysis of obtained results showed that the cross-linking density (υ_χ_) was higher for networks constructed of **COP** ^**8/2**^ than those based on **COP** ^**9/1**^ (Table 5) in accordance with the relationship υ_χ_ = *G*_N_/*R* × *T*,^35^ i.e., the higher *G*_N_, the higher the cross-linking of the network (Figure 4b). Moreover, the lower the amount of incorporated drug into the network, the higher the cross-linking degree observed.

**Table 5:** Comparison of the Network Strand Density (υ_χ_) Determined for Drug-Loaded Films Swollen with Deionized Water for 15 min

water-swollen drug-loaded film	network strand density (υ_χ_), mol/m^3^
**IC-POSS** ^**Ph**^ **_COP** ^**9/1**^ **_CLT** ^**2**^	17.1
**IC-POSS** ^**Ph**^ **_COP** ^**8/2**^ **_CLT** ^**2**^	119.2
**IC-POSS** ^**iBu**^ **_COP** ^**9/1**^ **_CLT** ^**2**^	1.3
**IC-POSS** ^**iBu**^ **_COP** ^**8/2**^ **_CLT** ^**2**^	486.4
**IC-POSS** ^**Ph**^ **_COP** ^**9/1**^ **_CLT** ^**4**^	6.6
**IC-POSS** ^**Ph**^ **_COP** ^**8/2**^ **_CLT** ^**4**^	209.7
**IC-POSS** ^**iBu**^ **_COP** ^**9/1**^ **_CLT** ^**4**^	2.1
**IC-POSS** ^**iBu**^ **_COP** ^**8/2**^ **_CLT** ^**4**^	5.1

Water-swollen films loaded with drug were deposited on porcine skin and immersed in simulated vaginal fluid to monitor their behavior in the target site of application for 24 and 48 h, respectively (Table 6). Networks characterized by a lower cross-linking density (Table 5), i.e., those constructed mainly of **COP** ^**9/1**^, adhered better to porcine skin than systems based on **COP** ^**8/2**^. Networks constructed from cross-linked cages with **COP** ^**8/2**^ required more time to form an adherent, well water-swollen network layer on the porcine skin. The exception, however, was a network based on **IC-POSS** ^**Ph**^ **_COP** ^**8/2**^ **_CLT** ^**4**^, which did not adhere well to skin, which strictly resulted from both its high cross-linking density (209.7 mol/m^3^) and very low swelling ability. Despite its incubation even for 48 h in simulated vaginal fluid, the network did not change, retaining its initial integrity.

**Table 6:**
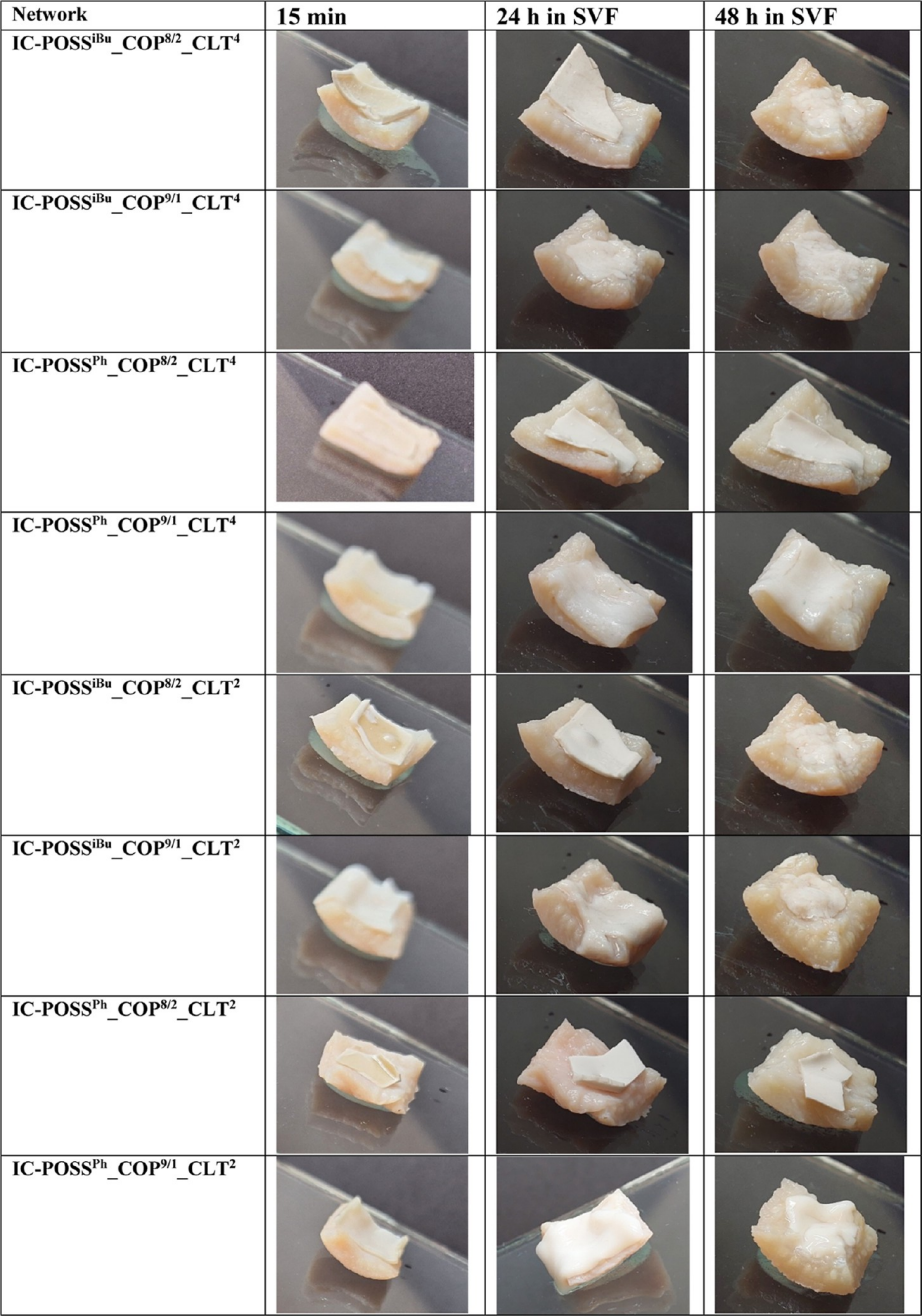
Visualization of Drug-Loaded Films Swollen with Water for 15 min Deposited on SVF-Saturated Porcine Skin after 24 and 48 h of Incubation in SVF

##### 3.3.4.2 Drug Release Profiles

In view of the intended vaginal application of the developed network-based films, we performed drug release profiles for clotrimazole-loaded networks in the simulated vaginal fluid at 37 °C.

The rate of drug release was lower for films made of the **COP** ^**8/2**^ copolymer, regardless of the amount of drug used (Figure 5). This behavior was related to the higher cross-linking density of the networks constructed from **COP** ^**8/2**^ in comparison to networks built of **COP** ^**9/1**^, which was confirmed for both dry and water-swollen networks (Figures 2 and 4b). Furthermore, the slower drug release rate observed for networks formed with **IC-POSS** ^**Ph**^ cages was correlated with their diminished swelling properties (Figure 4a). This behavior may indicate interactions between the aromatic rings of the drug molecules and the phenyl groups of the **IC-POSS** ^**Ph**^ cage. On the other hand, this phenomenon can be associated with a higher degree of network cross-linking resulting from the presence of additional, besides boronic esters, intermolecular interactions, i.e., π–π stacking between the phenyl groups of the **IC-POSS** ^**Ph**^ cages and drug molecules. These data explain the reduced segmental motion of the polymer chains that we observed in the DSC thermograms, even though a comparable molar fraction of boronic esters was formed in the networks constructed of both cage types.

**Figure 5:**
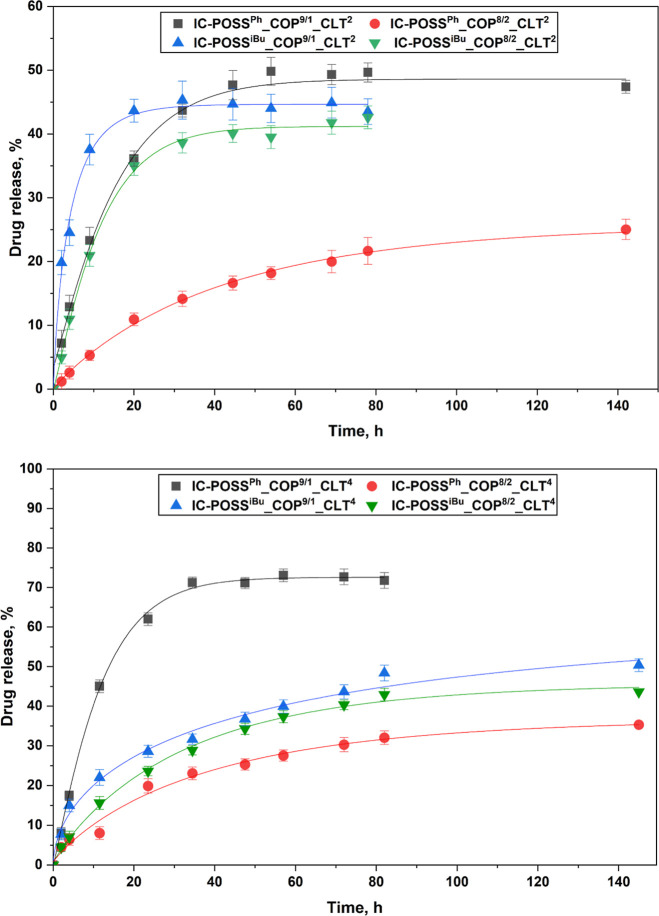
Comparison of clotrimazole release profiles obtained for film formulations with a molar ratio of clotrimazole to a cage equal to 2:1 (at the top) or 4:1 (at the bottom), respectively.

Experiments on drug release profiles revealed that the drug was released faster from networks in which a drug-to-cage ratio was 4:1 (Figure 5), characterized by the lowest *T*_g_ values for dry networks and low cross-linking density for water-swollen networks. Notably, a lower network cross-linking density results in a larger mesh size,^36,37^ i.e., the average distance between cross-links—which, in turn, facilitates faster active substance release. This reduced network density directly corresponds to increased network diffusivity.

The network-based films presented here demonstrate superior sustained drug release, reaching up to 80 h. In contrast, previously known polymeric carriers, such as those formulated with extruded poly­(ethylene oxide)^38^ or a chitosan/sodium carboxymethylcellulose/hydroxypropyl methylcellulose blend,^39^ complete their drug release within 8 h. These results show that our films have strong potential for long-term therapies lasting several days.

The drug release profiles obtained for all investigated film formulations were fitted to the Weibull model expressed by the following equation: $\frac{M}{M_{0}}=1-e^{\frac{{\left(t-T\right)}^{\beta }}{a}}$ , where *M* is the amount of drug dissolved as a function of time *t*, *M*_0_ is the total amount of drug being released, *T* accounts for the lag time measured as a result of the dissolution process, *a* is a scale parameter that describes the time dependence, and β describes the shape of the dissolution curve progression.^40^

β Shape parameter is used to determine the underlying drug release mechanism from drug carriers, enabling us to distinguish differences in the release mechanisms between our various formulations (Table 7). It was found that all material structure parameters—specifically, the molar fraction of the drug, the type of cage used, or the copolymer—affected the drug release mechanism. In the case of such formulations as **IC-POSS** ^**Ph**^ **_COP** ^**9/1**^ **_CLT** ^**2**^, **IC-POSS** ^**iBu**^ **_COP** ^**8/2**^ **_CLT** ^**2**^ and **IC-POSS** ^**Ph**^ **_COP** ^**9/1**^ **_CLT** ^**4**^, β > 1, which indicates that the drug release mechanism is associated with erosion or a burst drug release. For only one film formulation (**IC-POSS** ^**iBu**^ **_COP** ^**9/1**^ **_CLT** ^**4**^) β was below 0.75 which is indicative of a Fickian diffusion mechanism of drug release. For other film formulations, the shape parameter was in the range 0.75 < β < 1, which corresponds to a combined mechanism of drug release, i.e., the Fickian diffusion and swelling-controlled transport.

**Table 7:** β Shape Factor Values Determined for Clotrimazole Release Profiles Obtained for Investigated Film Formulations According to the Weibull Model

	Weibull model fitting	
film formulation	β	*R* ^2^
**IC-POSS** ^**Ph**^ **_COP** ^**9/1**^ **_CLT** ^**2**^	1.068 ± 0.132	0.995
**IC-POSS** ^**iBu**^ **_COP** ^**9/1**^ **_CLT** ^**2**^	0.803 ± 0.087	0.992
**IC-POSS** ^**Ph**^ **_COP** ^**8/2**^ **_CLT** ^**2**^	0.932 ± 0.044	0.998
**IC-POSS** ^**iBu**^ **_COP** ^**8/2**^ **_CLT** ^**2**^	1.162 ± 0.173	0.991
**IC-POSS** ^**Ph**^ **_COP** ^**9/1**^ **_CLT** ^**4**^	1.149 ± 0.122	0.998
**IC-POSS** ^**iBu**^ **_COP** ^**9/1**^ **_CLT** ^**4**^	0.563 ± 0.072	0.986
**IC-POSS** ^**Ph**^ **_COP** ^**8/2**^ **_CLT** ^**4**^	0.908 ± 0.067	0.996
**IC-POSS** ^**iBu**^ **_COP** ^**8/2**^ **_CLT** ^**4**^	0.875 ± 0.146	0.982

#### 3.3.5 Biological Investigations

##### 3.3.5.1 Antifungal Efficacy of Polymeric Constructs against Planktonic *C. albicans*

The antifungal efficacy of both drug-free and drug-loaded films was systematically evaluated against both *C. albicans* ATCC 10231 and SC5314 strains over 24 and 48 h (Table 8). For all tested formulations, CLT-loaded networks exhibited superior antifungal activity in comparison to free CLT, used in the same initial amount (400 μg/well), in view of the 50% drug release observed for networks after 24 and 48 h of incubation in SVF (Figure 5). Particularly, **IC-POSS** ^**iBu**^ **_COP** ^**8/2**^ **_CLT** ^**4**^ demonstrated the highest growth inhibition for both strains at 24 h (97.93% and 99.57%, respectively), maintaining robust efficacy at 48 h (97.53% and 99.41%). This performance exceeds that of CLT, especially for the SC5314 strain at 48 h (96.57%). These data indicate that the density of the **IC-POSS** ^**iBu**^ **_COP** ^**8/2**^ **_CLT** ^**4**^ network is crucial to enable the release of the drug in its molecular state, ensuring its greater activity. Therefore, a lower concentration of the drug released in its molecular state is needed to observe a therapeutic effect.

**Table 8:** Antifungal Activity of Open-POSS Cages, Drug-Free and Drug-Loaded Films against *C. albicans* ATCC 10231 and SC5314^a^

	growth inhibition (%)			
	*C. albicans* ATCC 10231		*C. albicans* SC5314	
film	24 h	48 h	24 h	48 h
control	0	0	0	0
**IC-POSS** ^**Ph**^ **_COP** ^**9/1**^ **_CLT** ^**2**^	96.30 ± 2.15***	88.33 ± 1.76***	98.38 ± 1.12***	79.48 ± 1.77***
**IC-POSS** ^**Ph**^ **_COP** ^**8/2**^ **_CTL** ^**2**^	96.88 ± 1.25***	95.08 ± 1.84***	99.44 ± 0.22***	98.77 ± 1.04***
**IC-POSS** ^**Ph**^ **_COP** ^**9/1**^ **_CTL** ^**4**^	93.86 ± 2.46***	81.98 ± 0.32***	91.34 ± 3.21***	80.01 ± 2.26***
**IC-POSS** ^**Ph**^ **_COP** ^**8/2**^ **_CTL** ^**4**^	97.78 ± 0.38***	95.93 ± 1.59***	99.48 ± 0.14***	98.33 ± 1.02***
**IC-POSS** ^**iBu**^ **_COP** ^**9/1**^ **_CLT** ^**2**^	97.64 ± 1.54***	96.91 ± 0.73***	98.86 ± 0.76***	94.89 ± 0.66***
**IC-POSS** ^**iBu**^ **_COP** ^**8/2**^ **_CLT** ^**2**^	97.28 ± 1.38***	97.30 ± 1.26***	99.38 ± 0.24***	99.02 ± 0.34***
**IC-POSS** ^**iBu**^ **_COP** ^**9/1**^ **_CLT** ^**4**^	95.55 ± 2.11***	94.12 ± 1.84***	96.81 ± 1.31***	91.92 ± 1.59***
**IC-POSS** ^**iBu**^ **_COP** ^**8/2**^ **_CLT** ^**4**^	97.93 ± 0.65***	97.53 ± 0.52***	99.57 ± 0.18***	99.41 ± 0.21***
**IC-POSS** ^**Ph**^ **_COP** ^**9/1**^	5.46 ± 2.11^ns^	6.72 ± 1.43^ns^	8.89 ± 2.19^ns^	10.76 ± 1.76^ns^
**IC-POSS** ^**Ph**^ **_COP** ^**8/2**^	8.63 ± 1.45^ns^	11.34 ± 1.74^ns^	8.99 ± 2.49^ns^	10.41 ± 2.15^ns^
**IC-POSS** ^**iBu**^ **_COP** ^**9/1**^	2.13 ± 0.43^ns^	10.34 ± 2.18^ns^	4.81 ± 1.74^ns^	5.17 ± 0.48^ns^
**IC-POSS** ^**iBu**^ **_COP** ^**8/2**^	1.30 ± 0.21^ns^	7.68 ± 1.64^ns^	1.54 ± 0.45^ns^	5.83 ± 1.44^ns^
CLT (400 μg/well; 800 μg/mL)	98.77 ± 1.15***	96.18 ± 0.71***	99.61 ± 0.22***	96.57 ± 0.76***

a The results are presented as the means ± SD of three independent experiments. ns indicates non-significance (*P* > 0.05), *0.05 > *P* > 0.01, **0.01 > *P* > 0.001, and ***P < 0.001.

This enhanced performance may be attributed to improved bioavailability of CLT facilitated by the network matrix, a phenomenon previously observed in nanoparticle-based drug delivery systems targeting fungal pathogens.^41,42^ The relatively low activity of unloaded networks (typically <10% inhibition) confirms that the observed antifungal effects are predominantly mediated by the released CLT, rather than inherent activity of the carrier system.

Interestingly, subtle structural differences in the network (i.e., POSS substituent: Ph vs iBu; and molar ratio of DMAM and 2-AAPBA: 9:1 vs 8:2) impacted antifungal efficacy. The networks based on **COP** ^**8/2**^ generally performed better than those constructed on **COP** ^**9/1**^, suggesting that the cross-linking density of the network affects the drug incorporation and then release dynamics.

##### 3.3.5.2 Time-Dependent Antifungal Activity

The sustained antifungal performance of the constructs was assessed over 10 days via the indirect measurement of growth inhibition mediated by the daily release of CLT (Table 9). Constructs such as **IC-POSS** ^**iBu**^ **_COP** ^**9/1**^ **_CLT** ^**4**^ and **IC-POSS** ^**iBu**^ **_COP** ^**8/2**^ **_CLT** ^**2**^ maintained significant activity at 240 h (75.10% and 71.99% inhibition, respectively, against *C. albicans* ATCC 10231), while others like **IC-POSS** ^**Ph**^ **_COP** ^**8/2**^ **_CLT** ^**2**^ declined more rapidly (16.34%).

**Table 9:** Antifungal Activity of CLT Released from Films after 24, 120, and 240 h of Incubation^a^

	growth inhibition (%)					
	*C. albicans* ATCC 10231			*C. albicans* SC5314		
film	24 h	120 h	240 h	24 h	120 h	240 h
control	0		0	0		0
**IC-POSS** ^**Ph**^ **_COP** ^**9/1**^ **_CLT** ^**2**^	99.54 ± 0.16***	83.22 ± 1.64***	70.62 ± 2.11**	99.70 ± 0.05***	82.47 ± 0.74***	72.66 ± 1.18**
**IC-POSS** ^**Ph**^ **_COP** ^**8/2**^ **_CTL** ^**2**^	97.29 ± 2.16***	52.28 ± 2.51**	16.34 ± 0.36^ns^	97.40 ± 0.72***	76.28 ± 2.17***	57.63 ± 2.42**
**IC-POSS** ^**Ph**^ **_COP** ^**9/1**^ **_CTL** ^**4**^	97.12 ± 0.14***	73.18 ± 1.15**	59.63 ± 2.16**	98.79 ± 0.55***	82.31 ± 0.84***	67.93 ± 1.53**
**IC-POSS** ^**Ph**^ **_COP** ^**8/2**^ **_CTL** ^**4**^	97.61 ± 0.17***	61.57 ± 2.03**	37.98 ± 0.47*	98.83 ± 1.11***	78.47 ± 1.73***	62.29 ± 0.59**
**IC-POSS** ^**iBu**^ **_COP** ^**9/1**^ **_CLT** ^**2**^	99.43 ± 0.04***	85.14 ± 0.48***	70.69 ± 0.46**	99.54 ± 0.26***	84.31 ± 1.48***	72.47 ± 1.82**
**IC-POSS** ^**iBu**^ **_COP** ^**8/2**^ **_CLT** ^**2**^	99.12 ± 0.35***	84.18 ± 1.63***	71.99 ± 2.19**	99.44 ± 0.17***	75.47 ± 0.34***	68.25 ± 1.77**
**IC-POSS** ^**iBu**^ **_COP** ^**9/1**^ **_CLT** ^**4**^	99.31 ± 0.19***	86.87 ± 0.26***	75.10 ± 1.86***	99.61 ± 0.16***	83.58 ± 2.51***	73.02 ± 0.94**
**IC-POSS** ^**iBu**^ **_COP** ^**8/2**^ **_CLT** ^**4**^	99.15 ± 0.14***	85.17 ± 0.48***	72.44 ± 0.79**	99.31 ± 0.29***	74.26 ± 0.27**	59.50 ± 0.41***
**IC-POSS** ^**Ph**^ **_COP** ^**9/1**^	4.62 ± 2.11^ns^	4.77 ± 0.13^ns^	5.04 ± 0.49^ns^	6.40 ± 0.93^ns^	6.48 ± 0.37^ns^	7.99 ± 0.49^ns^
**IC-POSS** ^**Ph**^ **_COP** ^**8/2**^	5.26 ± 0.52^ns^	5.69 ± 0.68^ns^	6.42 ± 0.41^ns^	7.79 ± 1.14^ns^	7.94 ± 1.31^ns^	8.68 ± 1.14^ns^
**IC-POSS** ^**iBu**^ **_COP** ^**9/1**^	3.37 ± 0.74^ns^	4.14 ± 0.36^ns^	5.41 ± 0.38^ns^	7.24 ± 0.58^ns^	7.54 ± 0.15^ns^	7.55 ± 2.17^ns^
**IC-POSS** ^**iBu**^ **_COP** ^**8/2**^	1.74 ± 0.45^ns^	2.36 ± 0.84^ns^	4.24 ± 1.25^ns^	6.04 ± 2.25^ns^	6.34 ± 0.31^ns^	7.01 ± 0.74^ns^

a The results are presented as the means ± SD of three independent experiments. ns indicates non-significance (*P* > 0.05), *0.05 > *P* > 0.01, **0.01 > *P* > 0.001, and ***P < 0.001.

This prolonged activity highlights the potential utility of these networks in the treatment of persistent infections. The performance of networks based on iBu-functionalized cages was notably more stable over time than that of their Ph analogs, which reflects differences in cross-linking density and thus swelling ability. Furthermore, this may also be the result of a stronger interaction between drug molecules and the phenyl groups of the cage than with the isobutyl groups, leading to a prolonged retention of the drug in networks constructed from cross-linked **IC-POSS** ^**Ph**^ cages,^34^ as observed in drug release experiments (Figure 4).

The antifungal performance varied slightly between *C. albicans* strains, with SC5314 generally exhibiting slightly higher resistance at later time points, aligning with known strain-dependent differences in antifungal susceptibility.^43^

##### 3.3.5.3 Activity against Mature *Candida* Biofilms

Treating preformed *Candida* biofilms is notably more challenging than targeting planktonic cells due to the protective matrix and altered fungal physiology. Remarkably, the CLT-loaded constructs substantially reduced the viability of mature biofilms after 24 h of treatment (Table 10). The **IC-POSS** ^**iBu**^ **_COP** ^**9/1**^ **_CLT** ^**4**^ construct exhibited the most pronounced antibiofilm effect, decreasing the metabolic activity of ATCC 10231 biofilms to 16.10%, slightly outperforming the free drug (CLT), which resulted in 18.59% viability.

**Table 10:** Effect of 24 h Treatment with CLT-Loaded Films on Pre-formed *Candida* Biofilms^a^

	biofilm metabolic activity (%)	
copolymer film	*C. albicans* ATCC 10231	*C. albicans* SC5314
control	100	100
**IC-POSS** ^**Ph**^ **_COP** ^**9/1**^ **_CLT** ^**2**^	21.61 ± 1.35***	30.61 ± 1.78**
**IC-POSS** ^**Ph**^ **_COP** ^**8/2**^ **_CTL** ^**2**^	18.53 ± 2.15***	30.12 ± 1.52**
**IC-POSS** ^**Ph**^ **_COP** ^**9/1**^ **_CTL** ^**4**^	23.68 ± 2.63***	31.30 ± 3.23**
**IC-POSS** ^**Ph**^ **_COP** ^**8/2**^ **_CTL** ^**4**^	20.46 ± 1.48***	31.62 ± 0.45**
**IC-POSS** ^**iBu**^ **_COP** ^**9/1**^ **_CLT** ^**2**^	18.70 ± 0.46***	34.51 ± 1.56**
**IC-POSS** ^**iBu**^ **_COP** ^**8/2**^ **_CLT** ^**2**^	20.07 ± 1.84***	31.96 ± 2.26**
**IC-POSS** ^**iBu**^ **_COP** ^**9/1**^ **_CLT** ^**4**^	16.10 ± 2.28***	28.90 ± 0.66**
**IC-POSS** ^**iBu**^ **_COP** ^**8/2**^ **_CLT** ^**4**^	21.44 ± 3.18***	30.44 ± 1.84
**IC-POSS** ^**Ph**^ **_COP** ^**9/1**^	99.24 ± 0.46^ns^	99.50 ± 1.72^ns^
**IC-POSS** ^**Ph**^ **_COP** ^**8/2**^	97.53 ± 2.13^ns^	96.74 ± 0.98^ns^
**IC-POSS** ^**iBu**^ **_COP** ^**9/1**^	88.13 ± 3.52^ns^	98.97 ± 1.03^ns^
**IC-POSS** ^**iBu**^ **_COP** ^**8/2**^	93.62 ± 1.59^ns^	97.70 ± 2.01^ns^
CLT (400 μg/well; 800 μg/mL)	18.59 ± 3.47***	29.74 ± 0.52**

a The results are presented as the means ± SD of three independent experiments. ns indicates non-significance (*P* > 0.05), *0.05 > *P* > 0.01, **0.01 > *P* > 0.001, and ***P < 0.001.

While all CLT-loaded films outperformed their unloaded counterparts, which maintained viability over 85%, there were still residual metabolic activities in the 30–35% range for SC5314 biofilms. This suggests that despite improved delivery, some biofilm subpopulations remain resistant or tolerant. This partial resistance is in line with previous studies reporting incomplete eradication of fungal biofilms by using conventional azoles. Of note, considering that in experiments on drug release after 24 h of incubation in SVF, less than 50% of the drug was released from the films, the improved antifungal performance of clotrimazole released from the films relative to free CLT suggests enhanced drug penetration or retention in the biofilm matrix, which is a key advantage for elaborated delivery systems.^44^

##### 3.3.5.4 Cytocompatibility with NHDF Cells

To ensure biocompatibility, the cytotoxicity of the films was assessed by using NHDF cells (Table 11). Drug-free networks exhibited negligible toxicity (>96% viability), confirming the inert nature of their building components. In contrast, CLT-loaded systems showed moderate cytotoxicity (71–85%), comparable to that of CLT (72.09%). The networks based on **IC-POSS** ^**iBu**^ cages again showed better tolerability, with the **IC-POSS** ^**iBu**^ **_COP** ^**8/2**^ **_CLT** ^**2**^ system achieving the highest viability (85.12%).

**Table 11:** Cytotoxicity of Drug-Free and CLT-Loaded Networks toward NHDF, Assessed by the MTT Assay after 24 h Exposure^a^

copolymer film	viability (%)
control	100
**IC-POSS** ^**Ph**^ **_COP** ^**9/1**^ **_CLT** ^**2**^	78.64 ± 1.25^ns^
**IC-POSS** ^**Ph**^ **_COP** ^**8/2**^ **_CTL** ^**2**^	79.05 ± 1.56^ns^
**IC-POSS** ^**Ph**^ **_COP** ^**9/1**^ **_CTL** ^**4**^	74.49 ± 0.83*
**IC-POSS** ^**Ph**^ **_COP** ^**8/2**^ **_CTL** ^**4**^	73.50 ± 1.58*
**IC-POSS** ^**iBu**^ **_COP** ^**9/1**^ **_CLT** ^**2**^	77.39 ± 0.87^ns^
**IC-POSS** ^**iBu**^ **_COP** ^**8/2**^ **_CLT** ^**2**^	85.12 ± 0.38^ns^
**IC-POSS** ^**iBu**^ **_COP** ^**9/1**^ **_CLT** ^**4**^	75.43 ± 1.58^ns^
**IC-POSS** ^**iBu**^ **_COP** ^**8/2**^ **_CLT** ^**4**^	71.28 ± 1.36*
**IC-POSS** ^**Ph**^ **_COP** ^**9/1**^	96.83 ± 1.74^ns^
**IC-POSS** ^**Ph**^ **_COP** ^**8/2**^	97.35 ± 0.68^ns^
**IC-POSS** ^**iBu**^ **_COP** ^**9/1**^	97.82 ± 1.82^ns^
**IC-POSS** ^**iBu**^ **_COP** ^**8/2**^	98.34 ± 1.81^ns^
CLT (400 μg/well; 800 μg/mL)	72.09 ± 1.31*

a The results are presented as the means ± SD of three independent experiments. ns indicates non-significance (*P* > 0.05), *0.05 > *P* > 0.01, **0.01 > *P* > 0.001, and ***P < 0.001.

This moderate cytotoxicity likely stems from CLT itself, rather than the carrier system, and is consistent with other reports of azole toxicity at similar concentrations in human cell lines.^45^ Nonetheless, viability above 70% is generally considered acceptable for topical applications or controlled-release devices, especially where local cytotoxicity can be offset by therapeutic efficacy.

## 4 Conclusions

In this work, we successfully developed and characterized novel water-swellable polymeric film formulations for enhanced clotrimazole delivery, specifically targeting vulvovaginal candidiasis and its associated *Candida* biofilms. Our innovative approach involved the reversible cross-linking of asymmetric hydrophilic–hydrophobic open-POSS cages equipped with phenyl or isobutyl groups (**IC-POSS** ^**Ph**^ or **IC-POSS** ^**iBu**^, respectively) with tailored poly­(dimethylacrylamide-2-acrylamidephenylboronic acid) and P­(DMAM-2-AAPBA) copolymers. We showed that **IC-POSS** ^**Ph**^ cages intrinsically prevented clotrimazole crystallization, likely due to π–π stacking interactions, ensuring a homogeneous drug distribution. For **IC-POSS** ^**iBu**^ cages, the judicious selection of P­(DMAM-2-AAPBA) copolymers was critical in achieving a uniform clotrimazole dispersion. By modulating the 2-AAPBA content in the copolymer, we precisely tuned the network’s cross-linking density. Higher cross-linking and strong drug–cage/cage–cage interactions (particularly in **IC-POSS** ^**Ph**^ systems) resulted in slower, more sustained clotrimazole release profiles, showcasing the ability to control drug delivery over time. Crucially, clotrimazole-loaded films exhibited significantly enhanced antifungal activity against both planktonic *C. albicans* strains and, importantly, against mature *Candida* biofilms, outperforming the free drug. This superior therapeutic outcome stems from the networks’ capacity to maintain clotrimazole in an amorphous state and facilitate its controlled, molecular-level release, thereby substantially improving its bioavailability at the infection site. The developed film formulations also demonstrated excellent cytocompatibility, which is of great importance for safe and effective vaginal applications.

Summing up, this work highlights how subtle structural modifications within the components building these novel networks are critical for achieving desired biological functions. The presented strategy for constructing tunable film formulations offers a significant advancement, applicable not only to targeted antifungal drug delivery but also more broadly to hydrophobic drug carriers across diverse biomedical therapies. These materials offer a promising route for developing more effective and patient-compliant treatments by overcoming solubility limitations and effectively combating biofilm-associated infections.

Long-term, controlled release of clotrimazole at a constant therapeutic concentration is crucial for maintaining effective antifungal activity and preventing recurrent infections.

## Supplementary Material



## Notes

^1^H, ^13^C, and ^29^Si NMR spectra, FTIR, MALDI-MS, and TGA data for open-POSS cages and their precursors, ^1^H and ^1^H DOSY NMR spectra for copolymers, and DSC thermograms recorded for drug-loaded networks in the second heating (PDF)

## References

[1] Sun Z., Ge X., Qiu B., Xiang Z., Jiang C., Wu J., Li Y. (2023). Vulvovaginal
candidiasis and vaginal microflora interaction: Microflora
changes and probiotic therapy. Front. Cell.
Infect. Microbiol..

[2] Parazzini F., Di Cintio E., Chiantera V., Guaschino S. (2000). Determinants
of different Candida species infections of the genital tract in women.
Sporachrom Study Geoup. Eur. J. Obstet. Gynecol.
Reprod. Biol..

[3] Jeanmonod, R. ; Chippa, V. ; Jeanmonod, D. Vaginal Candidiasis; StatPearls: Treasure Island (FL), 2025. Disclosure: Rebecca Jeanmonod declares no relevant financial relationships with ineligible companies. Disclosure: Venu Chippa declares no relevant financial relationships with ineligible companies. Disclosure: Donald Jeanmonod declares no relevant financial relationships with ineligible companies.

[4] Harriott M. M., Lilly E. A., Rodriguez T. E., Fidel P. L., Noverr M. C. (2010). Candida
albicans forms biofilms on the vaginal mucosa. Microbiology.

[5] Hassan Y., Chew S. Y., Than L. T. L. (2021). Candida glabrata: Pathogenicity and
Resistance Mechanisms for Adaptation and Survival. J. Fungi.

[6] Czechowicz P., Nowicka J., Gosciniak G. (2022). Virulence
Factors of Candida spp.
and Host Immune Response Important in the Pathogenesis of Vulvovaginal
Candidiasis. Int. J. Mol. Sci..

[7] Saadatfar F., Shayanfar A., Rahimpour E., Barzegar-Jalali M., Martinez F., Bolourtchian M., Jouyban A. (2018). Measurement and correlation
of clotrimazole solubility in ethanol plus water mixtures at = (293.2
to 313.2) K. J. Mol. Liq..

[8] McDermott A. (2022). Drug-resistant
fungi on the rise. Proc. Natl. Acad. Sci. U.S.A..

[9] Logan A., Wolfe A., Williamson J. C. (2022). Antifungal
Resistance and the Role
of New Therapeutic Agents. Curr. Infect. Dis.
Rep..

[10] Cui X., Wang L., Lu Y., Yue C. (2022). Development and research
progress of anti-drug resistant fungal drugs. J. Infect. Public Health.

[11] Gosecka M., Jaworska-Krych D., Gosecki M., Wielgus E., Marcinkowska M., Janaszewska A., Klajnert-Maculewicz B. (2022). Self-Healable,
Injectable Hydrogel
with Enhanced Clotrimazole Solubilization as a Potential Therapeutic
Platform for Gynecology. Biomacromolecules.

[12] Gosecka M., Gosecki M., Ziemczonek P., Urbaniak M., Wielgus E., Marcinkowska M., Janaszewska A., Klajnert-Maculewicz B. (2024). Selective
Anticervical Cancer Injectable and Self-Healable Hydrogel Platforms
Constructed of Drug-Loaded Cross-Linkable Unimolecular Micelles in
a Single and Combination Therapy. ACS Appl.
Mater. Interfaces.

[13] Gosecki M., Ziemczonek P., Gosecka M., Urbaniak M., Wielgus E., Marcinkowska M., Janaszewska A., Klajnert-Maculewicz B. (2023). Cross-linkable
star-hyperbranched unimolecular micelles for the enhancement of the
anticancer activity of clotrimazole. J. Mater.
Chem. B.

[14] Jaworska-Krych D., Gosecka M., Gosecki M., Urbaniak M., Dzitko K., Ciesielska A., Wielgus E., Kadlubowski S., Kozanecki M. (2024). Enhanced Solubility
and Bioavailability of Clotrimazole
in Aqueous Solutions with Hydrophobized Hyperbranched Polyglycidol
for Improved Antifungal Activity. ACS Appl.
Mater. Interfaces.

[15] Nematpour N., Moradipour P., Zangeneh M. M., Arkan E., Abdoli M., Behbood L. (2020). The application of nanomaterial science in the formulation
a novel antibiotic: Assessment of the antifungal properties of mucoadhesive
clotrimazole loaded nanofiber versus vaginal films. Mater. Sci. Eng., C.

[16] Notario-Pérez F., Cazorla-Luna R., Martín-Illana A., Galante J., Ruiz-Caro R., das Neves J., Veiga M. D. (2020). Design, fabrication
and characterisation of drug-loaded vaginal films: State-of-the-art. J. Controlled Release.

[17] Tejada G., Lamas M. C., Svetaz L., Salomón C. J., Alvarez V. A., Leonardi D. (2018). Effect of drug incorporation
technique
and polymer combination on the performance of biopolymeric antifungal
buccal films. Int. J. Pharm..

[18] Bassi P., Kaur G. (2017). Polymeric films as
a promising carrier for bioadhesive drug delivery:
Development, characterization and optimization. Saudi Pharm. J..

[19] das
Neves J., Sarmento B. (2017). Antiretroviral drug-loaded nanoparticles-in-films:
a new option for developing vaginal microbicides?. Expert Opin. Drug Delivery.

[20] Real D. A., Martinez M. V., Frattini A., Soazo M., Luque A. G., Biasoli M. S., Salomon C. J., Olivieri A. C., Leonardi D. (2013). Design, characterization,
and in vitro evaluation of antifungal polymeric films. AAPS PharmSciTech.

[21] Zhang W., Parniak M. A., Sarafianos S. G., Cost M. R., Rohan L. C. (2014). Development
of a vaginal delivery film containing EFdA, a novel anti-HIV nucleoside
reverse transcriptase inhibitor. Int. J. Pharm..

[22] Nakano T., Okamoto K., Imoto H., Naka K. (2023). Double-cyclopolymerization
using trifunctional incompletely condensed cage silsesquioxane with
methacryloyl groups. Polym. J..

[23] Ueda Y., Imoto H., Okada A., Xu H. Z., Yamane H., Naka K. (2021). Hybrid polyurethanes
composed of isobutyl-substituted open-cage silsesquioxane
in the main chains: synthesis, properties and surface segregation
in a polymer matrix. Polym. Chem..

[24] Tanaka R., Igarashi A., Hayashi T., Masunaga H., Takagi H., Shimizu N., Igarashi N., Sakurai S., Imoto H., Naka K. (2023). Corner-opened cage-silsesquioxane
as a directional template for tripodal
poly­(methyl methacrylate). Polym. Chem..

[25] Chen B. X., Lin X. L., Yang M. J., You Z. L., Liu W. F., Meng H. L., Zhou Y. H., Yuan H., Liao J. W. (2022). Engineered
partially open-cage fluorinated polyhedral oligomeric silsesquioxane
hybrid nanoparticle aggregates for surfaces with super-repellency
to widespread liquids. J. Mater. Chem. A.

[26] Imoto H., Katoh R., Naka K. (2018). Open-cage
silsesquioxane necklace
polymers having closed-cage silsesquioxane pendants. Polym. Chem..

[27] Deng C. C., Brooks W. L. A., Abboud K. A., Sumerlin B. S. (2015). Boronic Acid-Based
Hydrogels Undergo Self-Healing at Neutral and Acidic pH. ACS Macro Lett..

[28] Yuasa S., Sato Y., Imoto H., Naka K. (2018). Fabrication
of composite
films with poly­(methyl methacrylate) and incompletely condensed cage-silsesquioxane
fillers. J. Appl. Polym. Sci..

[29] Gillum A. M., Tsay E. Y., Kirsch D. R. (1984). Isolation of the Candida albicans
gene for orotidine-5′-phosphate decarboxylase by complementation
of S. cerevisiae ura3 and E. coli pyrF mutations. Mol. Gen. Genet..

[30] Janek T., Drzymala K., Dobrowolski A. (2020). In vitro efficacy of the lipopeptide
biosurfactant surfactin-C(15) and its complexes with divalent counterions
to inhibit Candida albicans biofilm and hyphal formation. Biofouling.

[31] Janek T., Krasowska A., Radwanska A., Lukaszewicz M. (2013). Lipopeptide
biosurfactant pseudofactin II induced apoptosis of melanoma A 375
cells by specific interaction with the plasma membrane. PLoS One.

[32] Paul, C. ; Hiemenz, T. P. L. Polymer Chemistry; CRC Press Taylor & Francis Group, 2007.

[33] Pal, K. ; Banerjee, I. Polymeric Gels: Characterization, Properties and Biomedical Applications; Woodhead Publishing: Cambridge, MA, 2018.

[34] Mei B. C., Lin T. W., Sheridan G. S., Evans C. M., Sing C. E., Schweizer K. S. (2023). How Segmental
Dynamics and Mesh Confinement Determine
the Selective Diffusivity of Molecules in Cross-Linked Dense Polymer
Networks. ACS Cent. Sci..

[35] Feldman K. E., Kade M. J., Meijer E. W., Hawker C. J., Kramer E. J. (2011). Model Transient
Networks from Strongly Hydrogen-Bonded Polymers. Macromolecules.

[36] Karvinen J., Ihalainen T. O., Calejo M. T., Jönkkäri I., Kellomäki M. (2019). Characterization
of the microstructure of hydrazone
crosslinked polysaccharide-based hydrogels through rheological and
diffusion studies. Mater. Sci. Eng., C.

[37] Kopac T., Rucigaj A., Krajnc M. (2020). The mutual
effect of the crosslinker
and biopolymer concentration on the desired hydrogel properties. Int. J. Biol. Macromol..

[38] Sawant K., Elkanayati R. M., Almotairy A., Repka M. A., Almutairi M. (2025). Clotrimazole
mucoadhesive films with extended-release properties for vaginal candidiasis-A
hot-melt extrusion application. J. Pharm. Sci..

[39] Sudeendra B. R., Umme H., Gupta R. K., Shivakumar H. G. (2010). Development
and characterization of bioadhesive vaginal films of clotrimazole
for vaginal candidiasis. Acta Pharm. Sci..

[40] Dash S., Murthy P. N., Nath L., Chowdhury P. (2010). Kinetic modeling
on drug release from controlled drug delivery systems. Acta Pol. Pharm..

[41] Nagasa G. D., Belete A. (2022). Review on Nanomaterials and Nano-Scaled Systems for
Topical and Systemic Delivery of Antifungal Drugs. J. Multidiscip. Healthc..

[42] Guo Y. X., He Y. X. (2024). Nanoparticle-Based Drug Delivery Systems: An Updated Strategy for
Treating Fungal Keratitis. Colloid Interface
Sci. Commun..

[43] Hacioglu M., Guzel C. B., Savage P. B., Tan A. S. B. (2019). Antifungal susceptibilities,
in vitro production of virulence factors and activities of ceragenins
against Candida spp. isolated from vulvovaginal candidiasis. Med. Mycol..

[44] Velazco-Medel M. A., Camacho-Cruz L. A., Lugo-González J. C., Bucio E. (2021). Antifungal
Polymers for Medical Applications. Med. Devices
Sens..

[45] Bolla P. K., Meraz C. A., Rodriguez V. A., Deaguero I., Singh M., Yellepeddi V. K., Renukuntla J. (2019). Clotrimazole Loaded Ufosomes for
Topical Delivery: Formulation Development and In-Vitro Studies. Molecules.

