# Supplementary material for: Reversibly Cross-Linked Asymmetric Hybrid Open-Polysilsesquioxane Films Enhancing Clotrimazole Bioavailability and Anti-Candida Mature Biofilm Activity for Vaginal Therapy
Source: ACS Appl Mater Interfaces. 2025 Oct 7;17(41):56730–48. doi: 10.1021/acsami.5c12791 (PMC12532088; doi:10.1021/acsami.5c12791)
Supplement: Supplementary file 1 [file am5c12791_si_001.pdf]

Supporting Information

Reversibly cross-linked asymmetric hybrid open-polysilsesquioxane films enhancing clotrimazole bioavailability and anti-*Candida* mature biofilm activity for vaginal therapy

Marta Madej-Gajewska,[a] Tomasz Janek,[b] Monika Gosecka,[c,*], Mateusz Gosecki,[c] Małgorzata Urbaniak,[c] Ewelina Wielgus,[c] Łukasz John[a,*]

[a]*Faculty of Chemistry, University of Wrocław, 14 F. Joliot-Curie, 50-383 Wrocław, Poland*

[b]*Department of Biotechnology and Food Microbiology, Wrocław University of Environmental and Life Sciences, 37 Chełmońskiego, 51-630 Wrocław, Poland*

[c]*Centre of Molecular and Macromolecular Studies, Polish Academy of Sciences, Sienkiewicza 112, 90-363 Łódź, Poland*

email addresses: mdybko@cbmm.lodz.pl

lukasz.john@uwr.edu.pl

1. Hepta(isobutyl)tris(dimethylsiloxy)-POSS

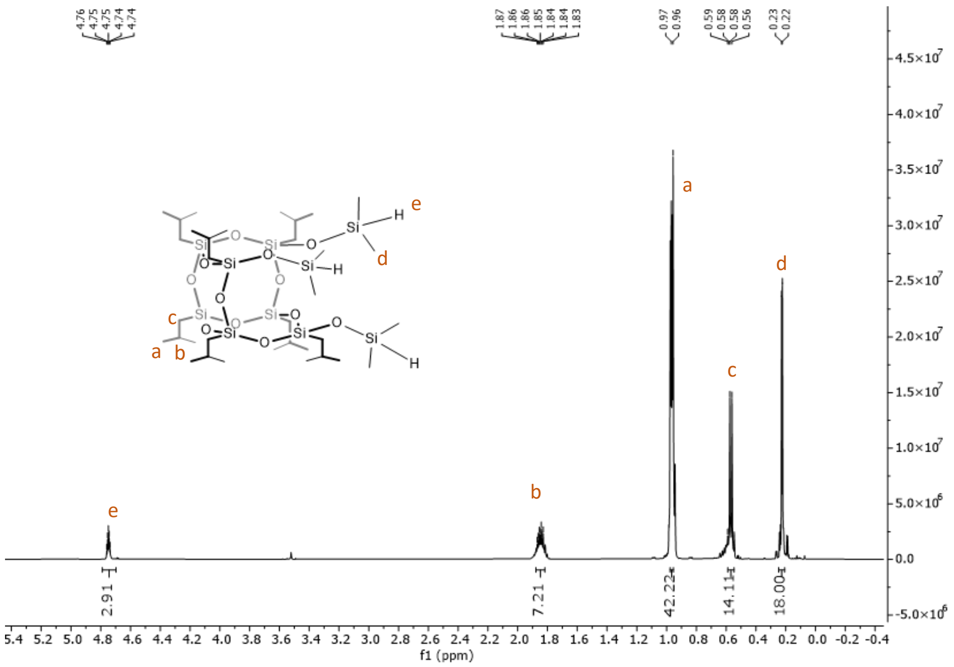

Figure S1. ^{1}H NMR spectra of hepta(isobutyl)tris(dimethylsiloxy)-POSS (500 MHz, $CDCl_3$, 300 K).

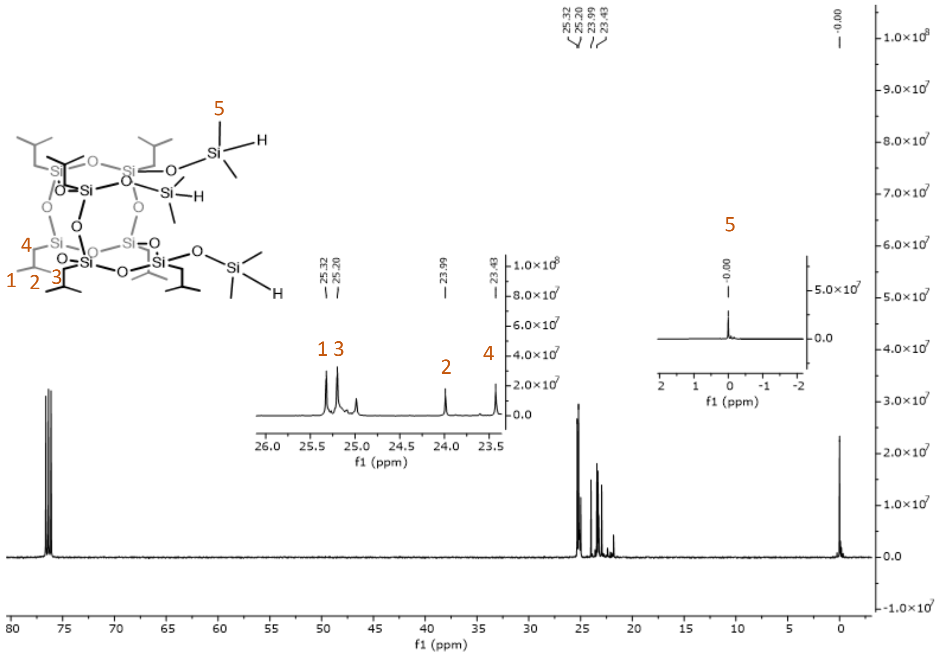

Figure S2. ^{13}C NMR spectra of hepta(isobutyl)tris(dimethylsiloxy)-POSS (126 MHz, $CDCl_3$, 300 K).

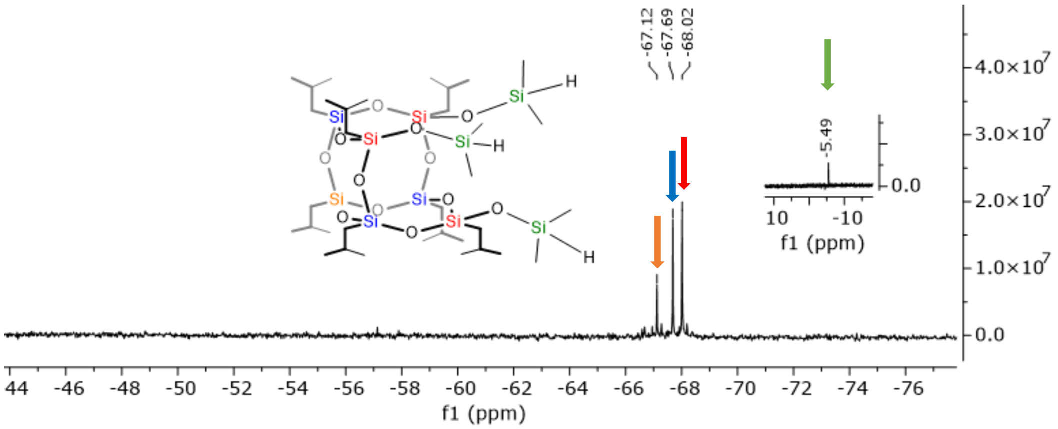

Figure S3. ^{29}Si NMR spectra of hepta(isobutyl)tris(dimethylsiloxy)-POSS (99 MHz, $CDCl_3$, 300 K).

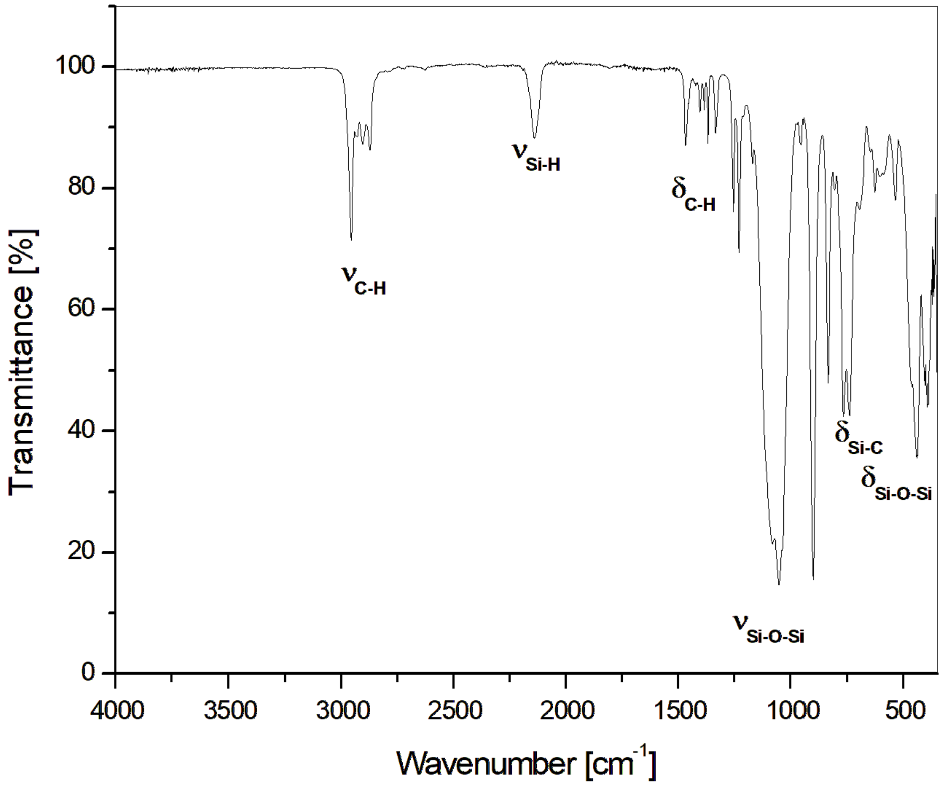

Figure S4. FT-IR spectrum of hepta(isobutyl)tris(dimethylsiloxy)-POSS (diamond ATR attachment).

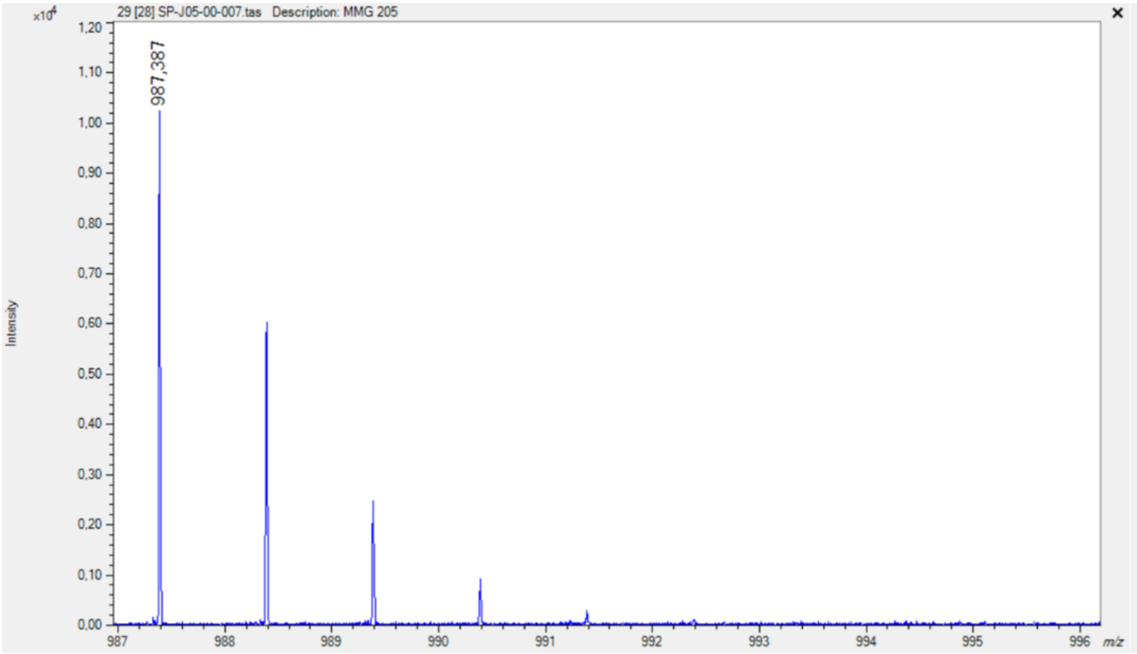

Figure S5. MALDI-MS of hepta(isobutyl)tris(dimethylsiloxy)-POSS: *m/z*: 987.387 {calc. $[M + Na]^+$ 987.36}.

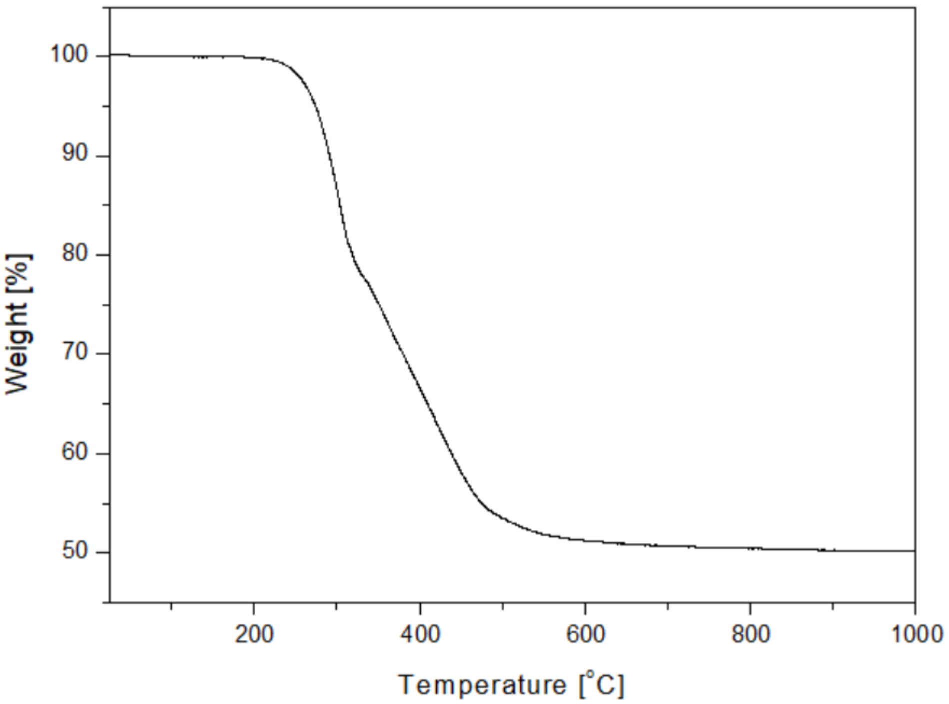

Figure S6. TGA curve of hepta(isobutyl)tris(dimethylsiloxy)-POSS ($\Delta T_{10\%}$ = 291.4 °C).

2. Hepta(phenyl)tris(dimethylsiloxy)-POSS

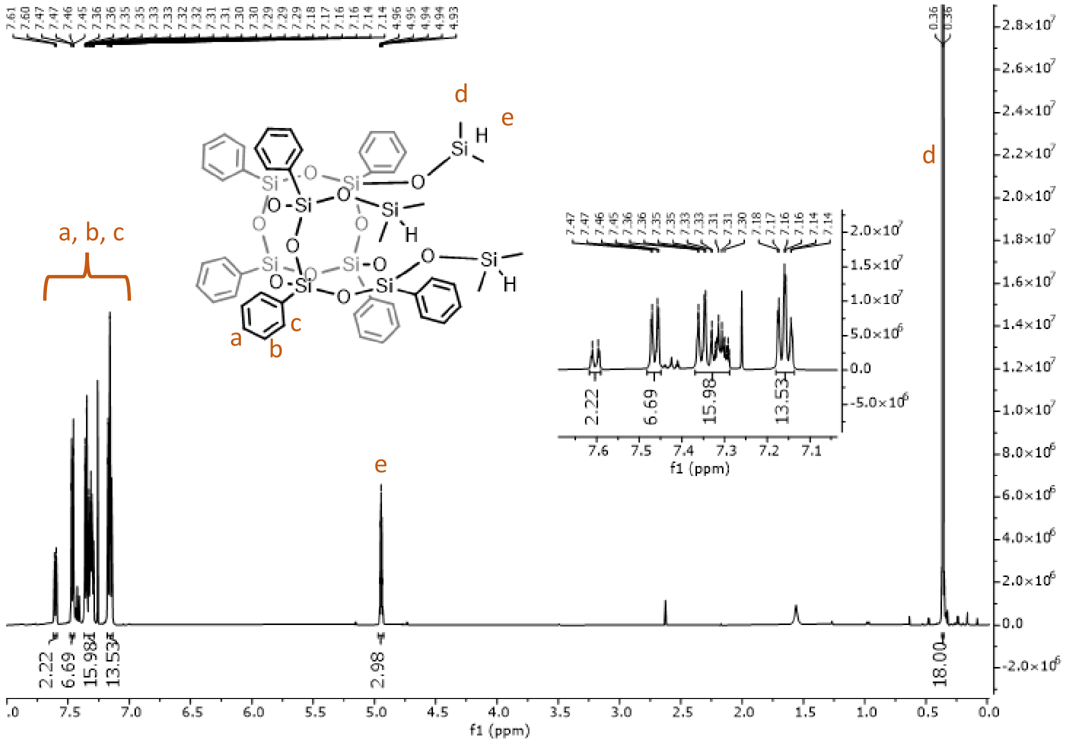

Figure S7. ^{1}H NMR spectra of hepta(phenyl)tris(dimethylsiloxy)-POSS (500 MHz, $CDCl_3$, 300 K).

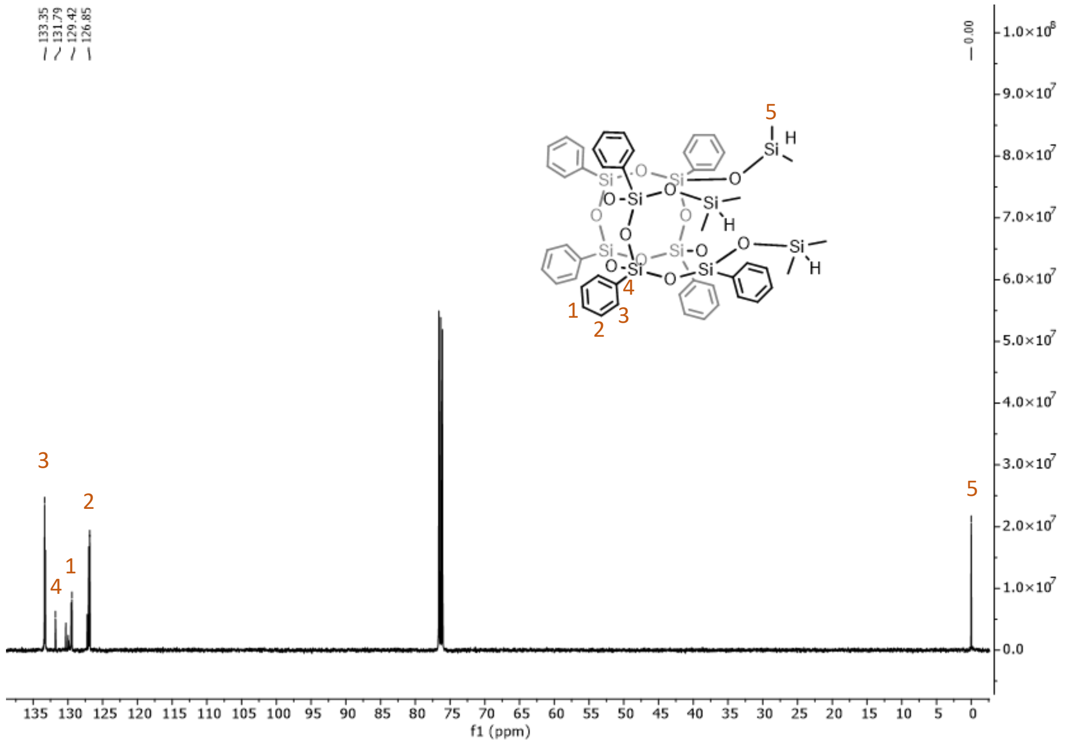

Figure S8. ^{13}C NMR spectra of hepta(phenyl)tris(dimethylsiloxy)-POSS (126 MHz, $CDCl_3$, 300 K).

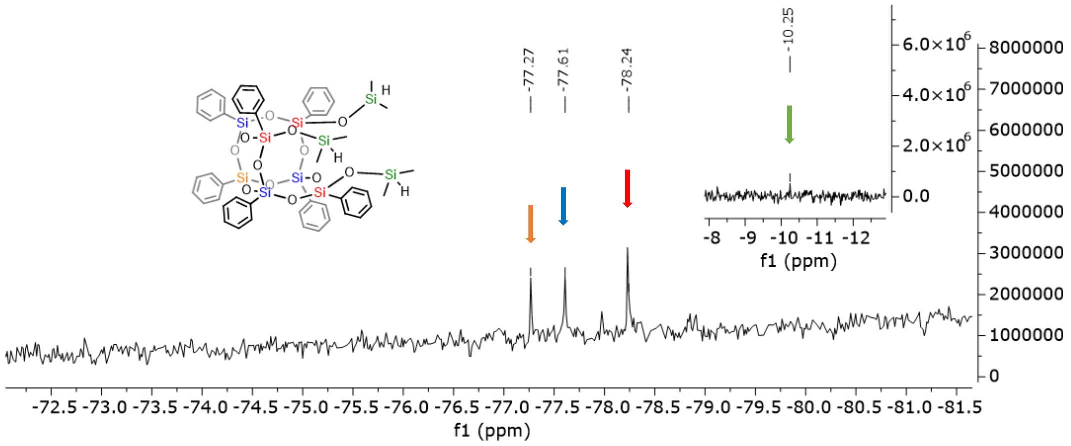

Figure S9. ^{29}Si NMR spectra of hepta(phenyl)tris(dimethylsiloxy)-POSS (99 MHz, $CDCl_3$, 300 K).

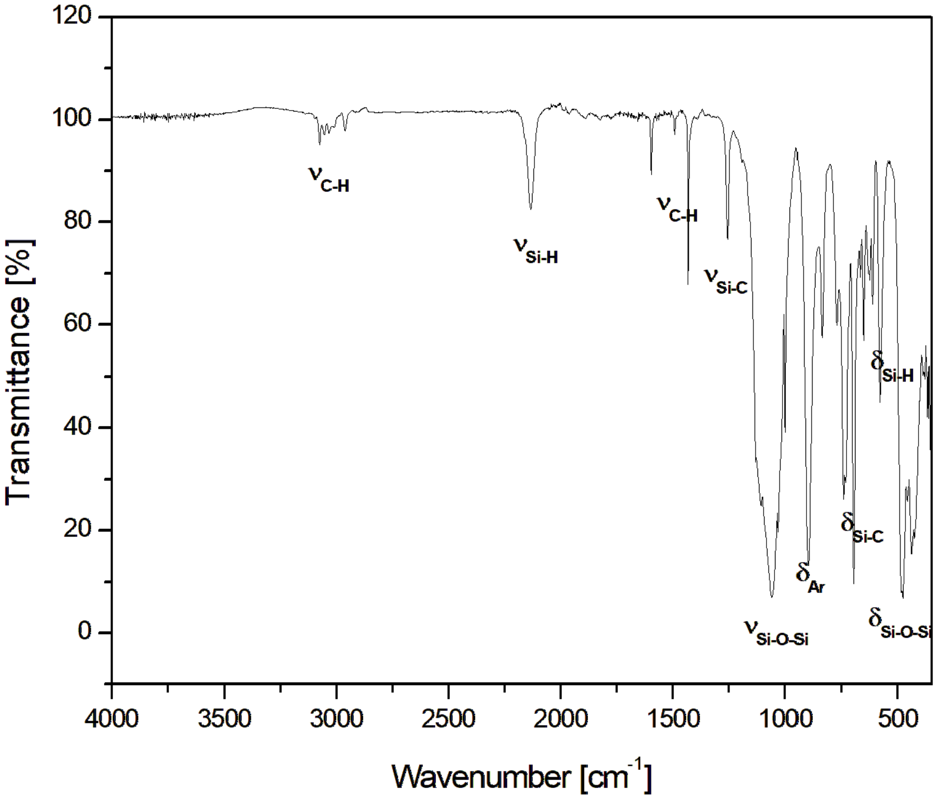

Figure S10. FT-IR spectrum of hepta(phenyl)tris(dimethylsiloxy)-POSS (diamond ATR attachment).

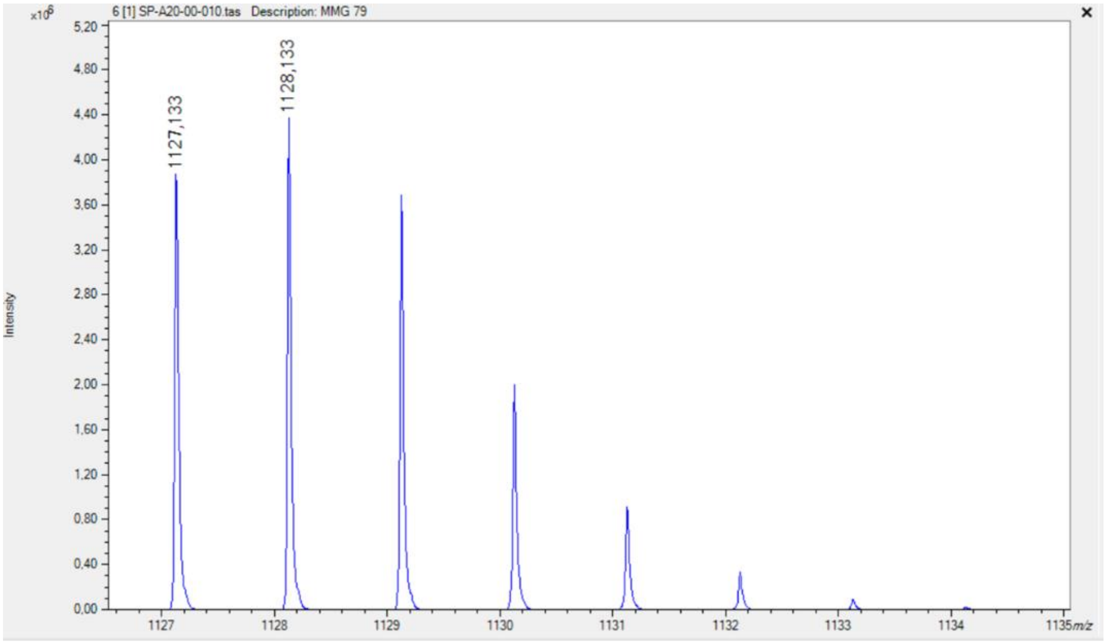

Figure S11. MALDI-MS of hepta(phenyl)tris(dimethylsiloxy)-POSS: *m/z*: 1127.133 {calc. $[M + Na]^+$ 1127.14}.

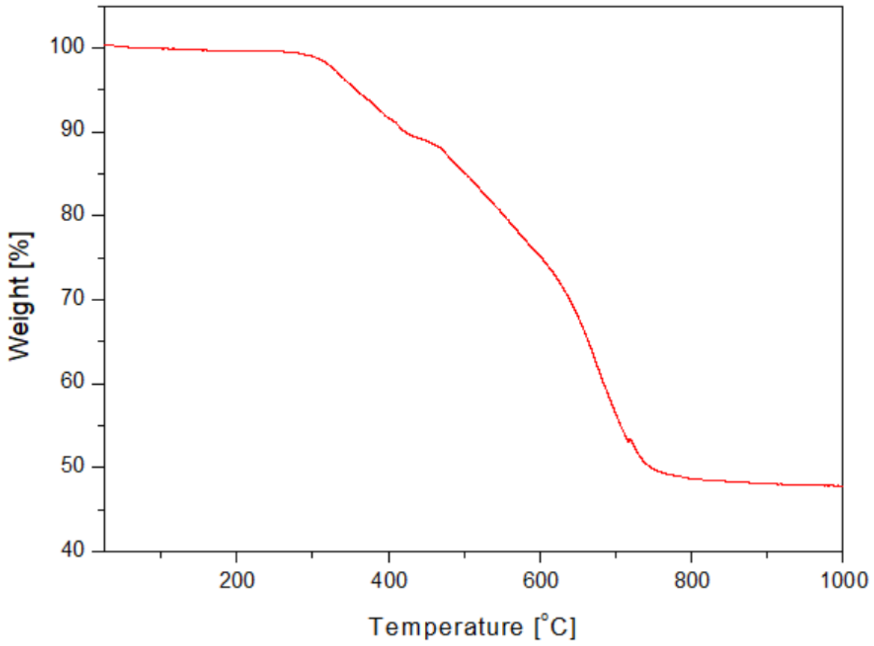

Figure S12. TGA curve of hepta(phenyl)tris(dimethylsiloxy)-POSS ($\Delta T_{10\%}$ = 422.2 °C).

3. IC-POSS$^{i\text{-}Bu}$

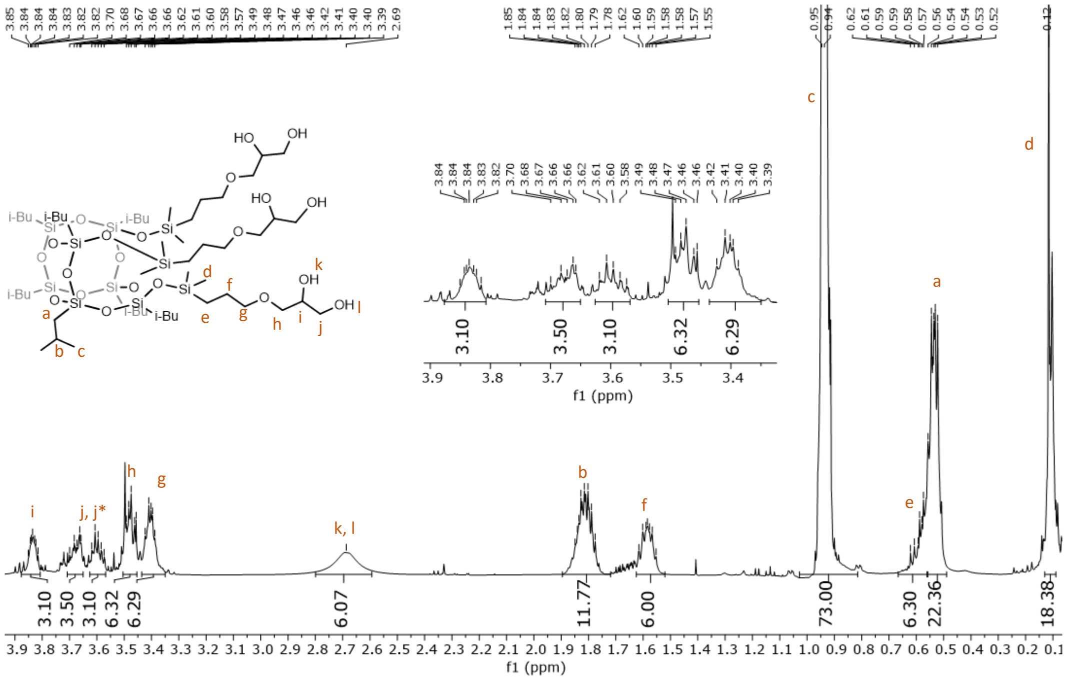

Figure S13. ^{1}H NMR spectra of IC-POSS^{iBu} (500 MHz, $CDCl_3$, 300 K).

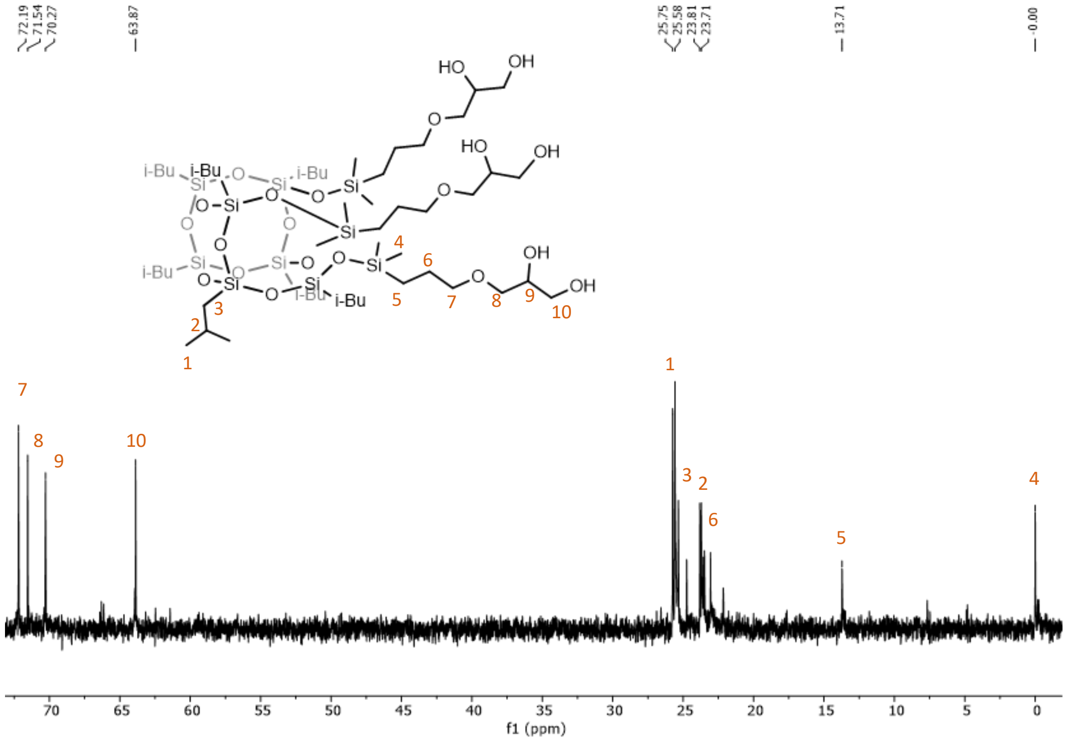

Figure S14. ^{13}C NMR spectra of IC-POSS^{iBu} (126 MHz, $CDCl_3$, 300 K).

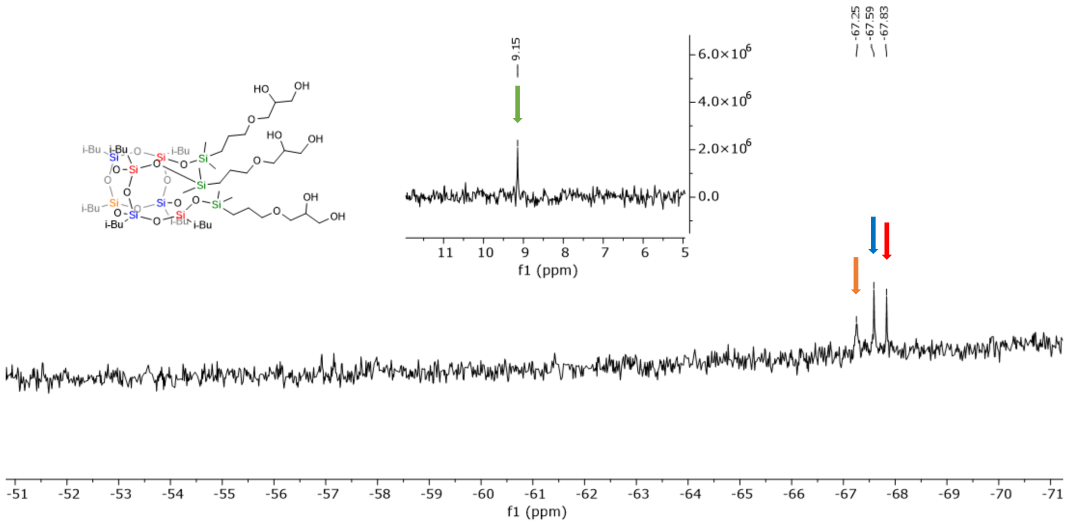

Figure S15. ^{29}Si NMR spectra of IC-POSS^{iBu} (99 MHz, $CDCl_3$, 300 K).

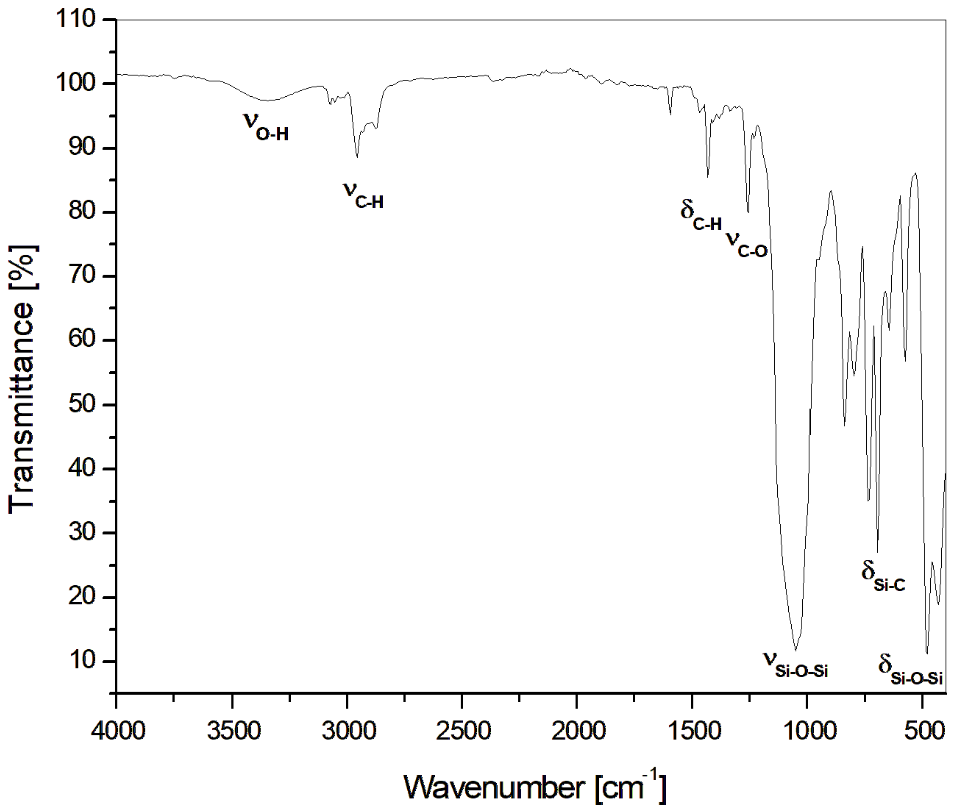

Figure S16. FT-IR spectrum of IC-POSS^{iBu} (diamond ATR attachment).

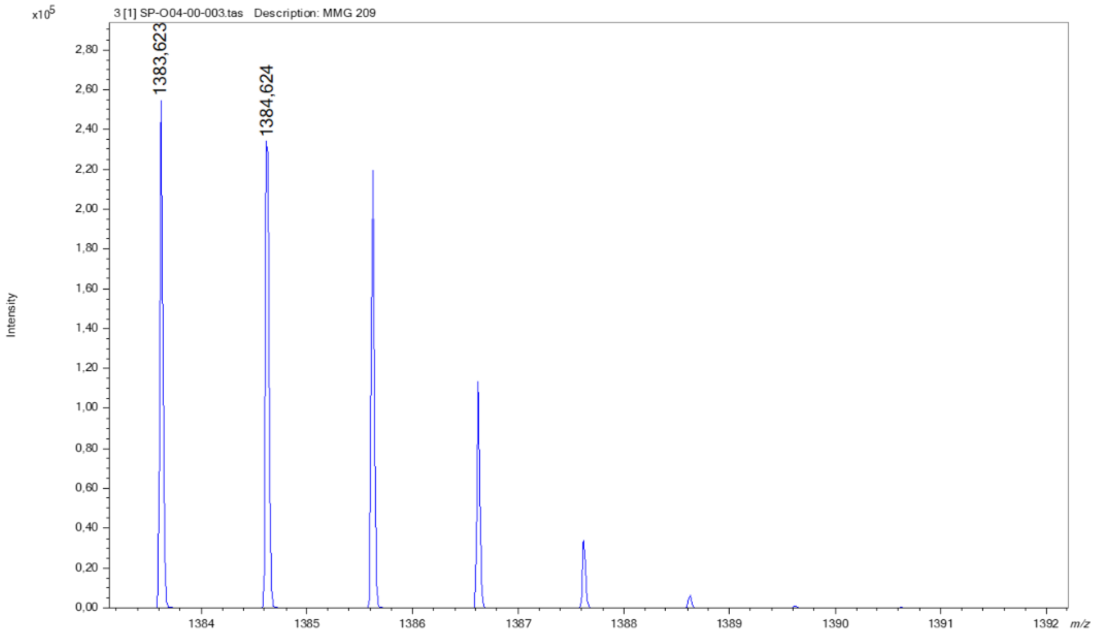

Figure S17. MALDI-MS of IC-POSS^{iBu}: *m/z*: 1383.62 {calc. $[M + Na]^+$ 1383.59}.

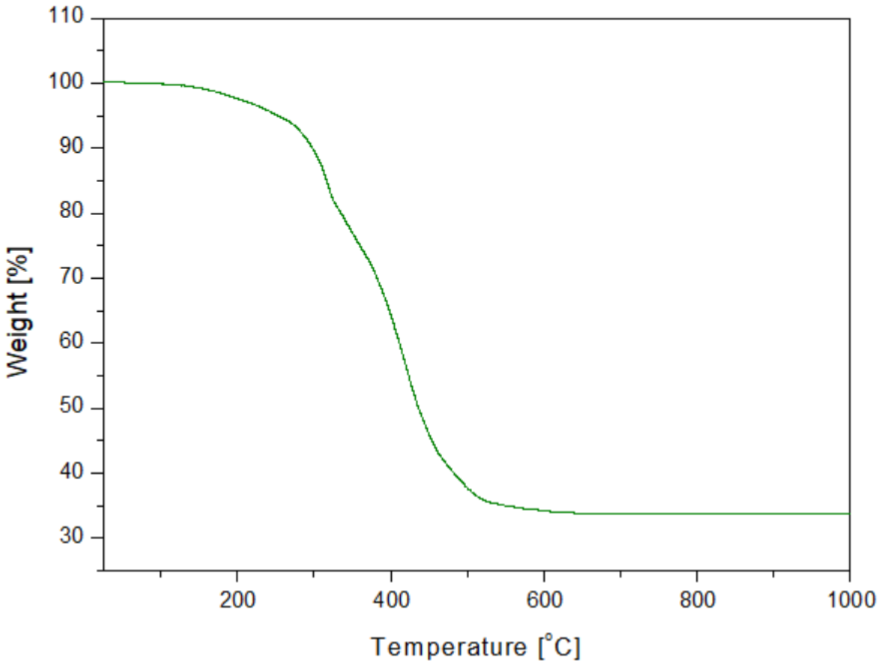

Figure S18. TGA curve of IC-POSS^{iBu} ($\Delta T_{10\%}$=297,8 °C).

4. IC-POSS^{Ph}

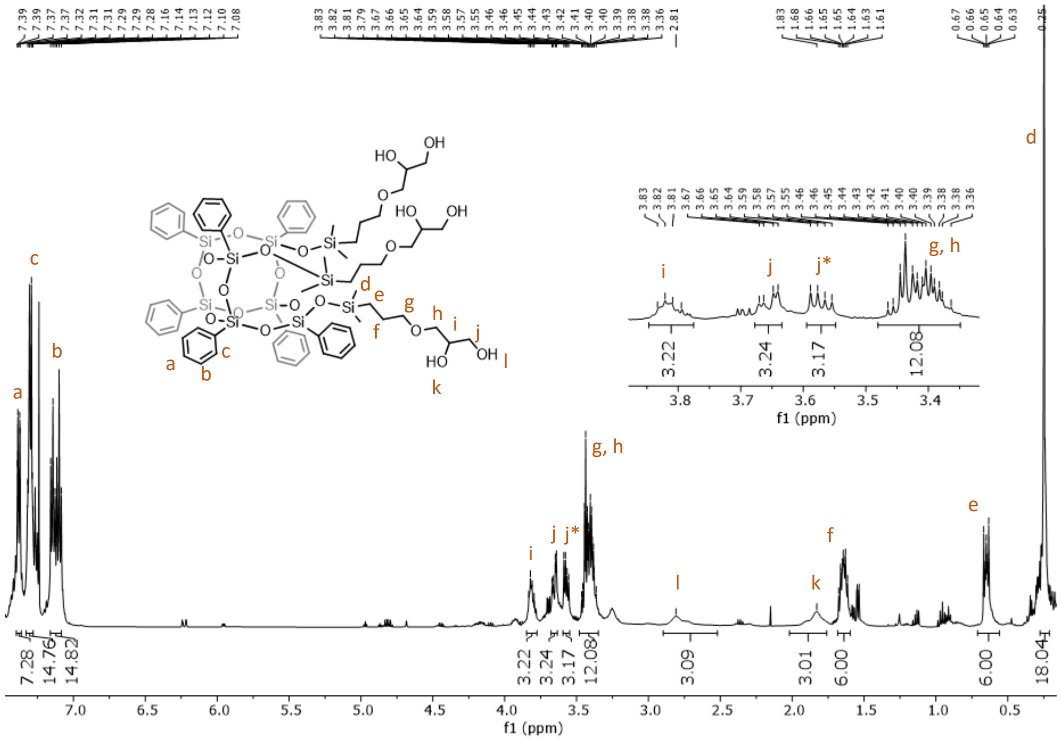

Figure S19. ^{1}H NMR spectra of IC-POSS^{Ph} (500 MHz, $CDCl_3$, 300 K).

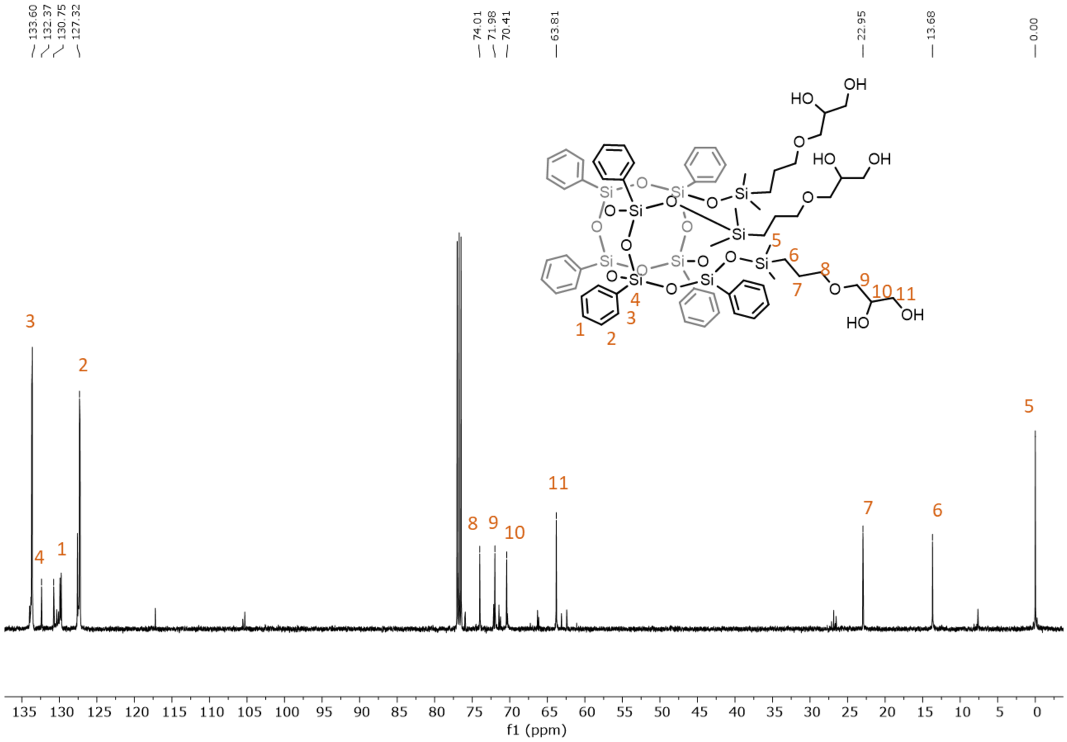

Figure S20. ^{13}C NMR spectra of IC-POSS^{Ph} (126 MHz, $CDCl_3$, 300 K).

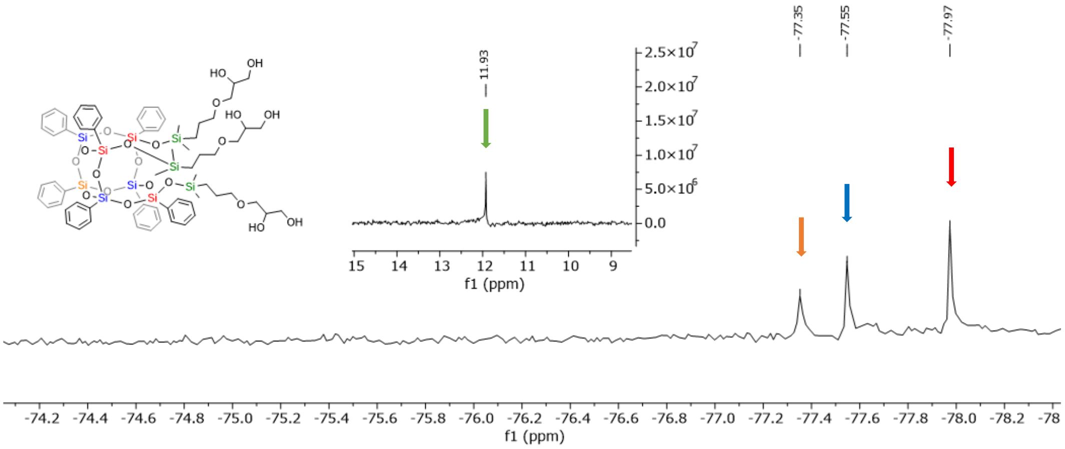

Figure S21. ^{29}Si NMR spectra of IC-POSS^{Ph} (99 MHz, $CDCl_3$, 300 K).

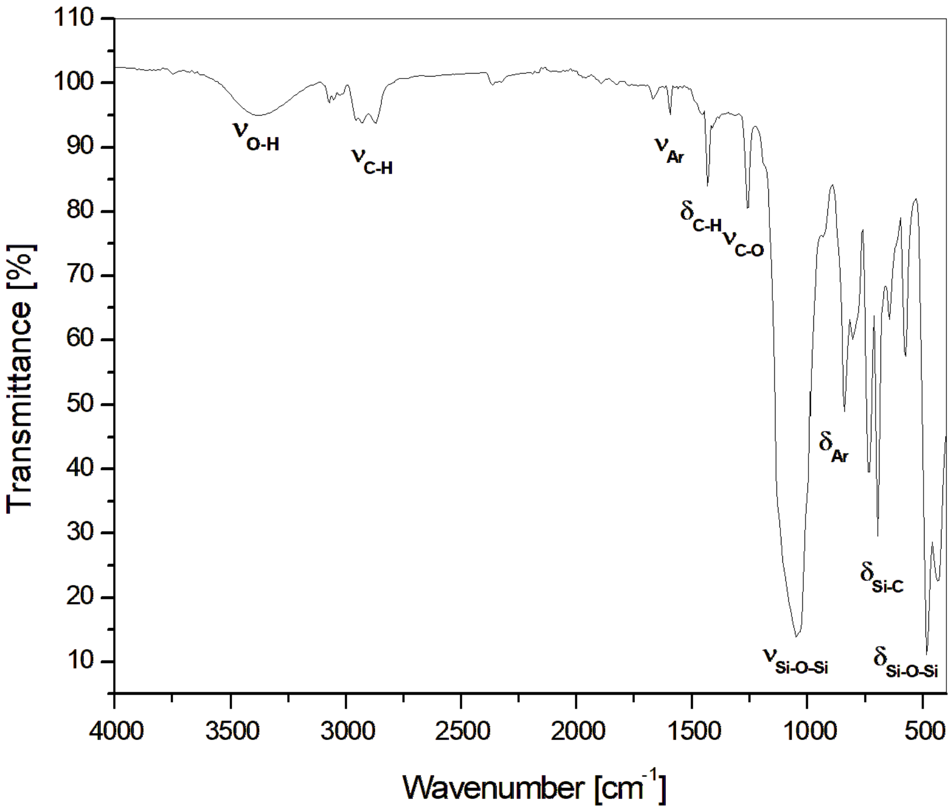

Figure S22. FT-IR spectrum of IC-POSS^{Ph} (diamond ATR attachment).

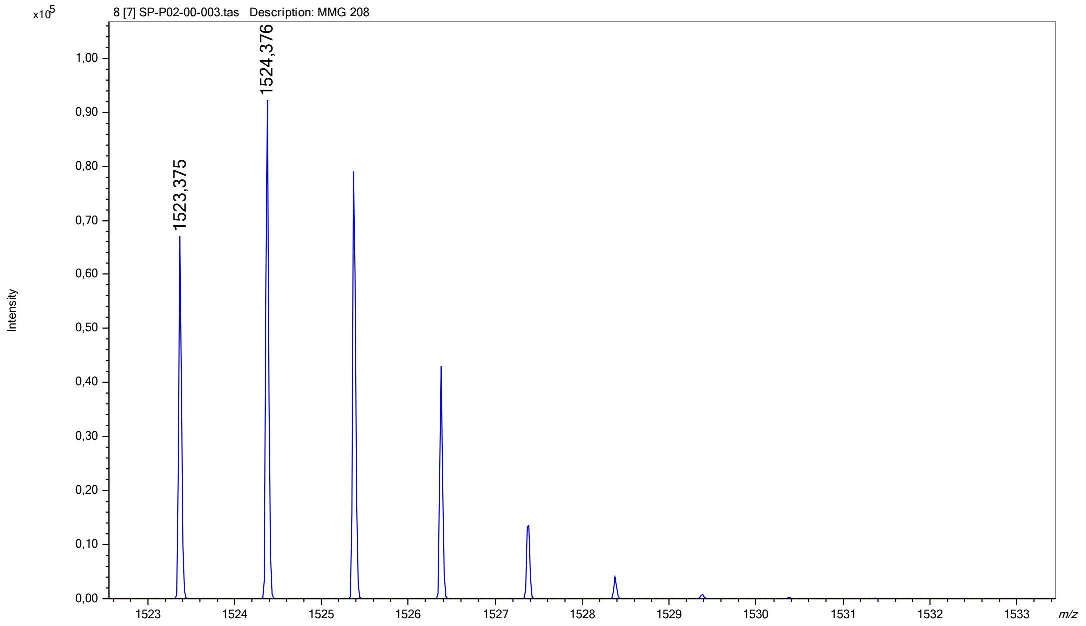

Figure S23. MALDI-MS of IC-POSS^{Ph}: *m/z:* 1523.38 {calc. [M + Na]$^+$ 1523.37}.

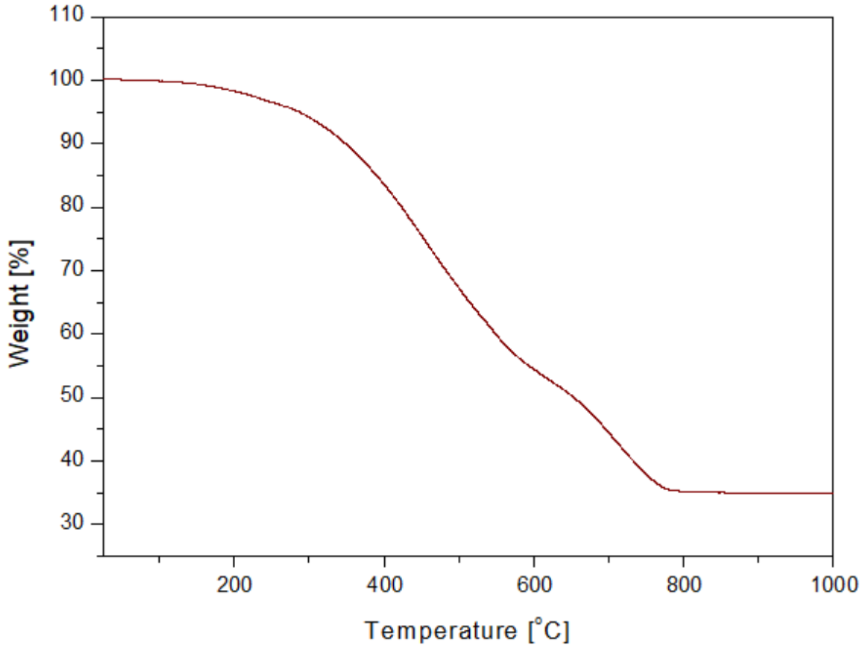

Figure S24. TGA curve of IC-POSS^{Ph} ($\Delta T_{10\%}$ = 348,6 °C).

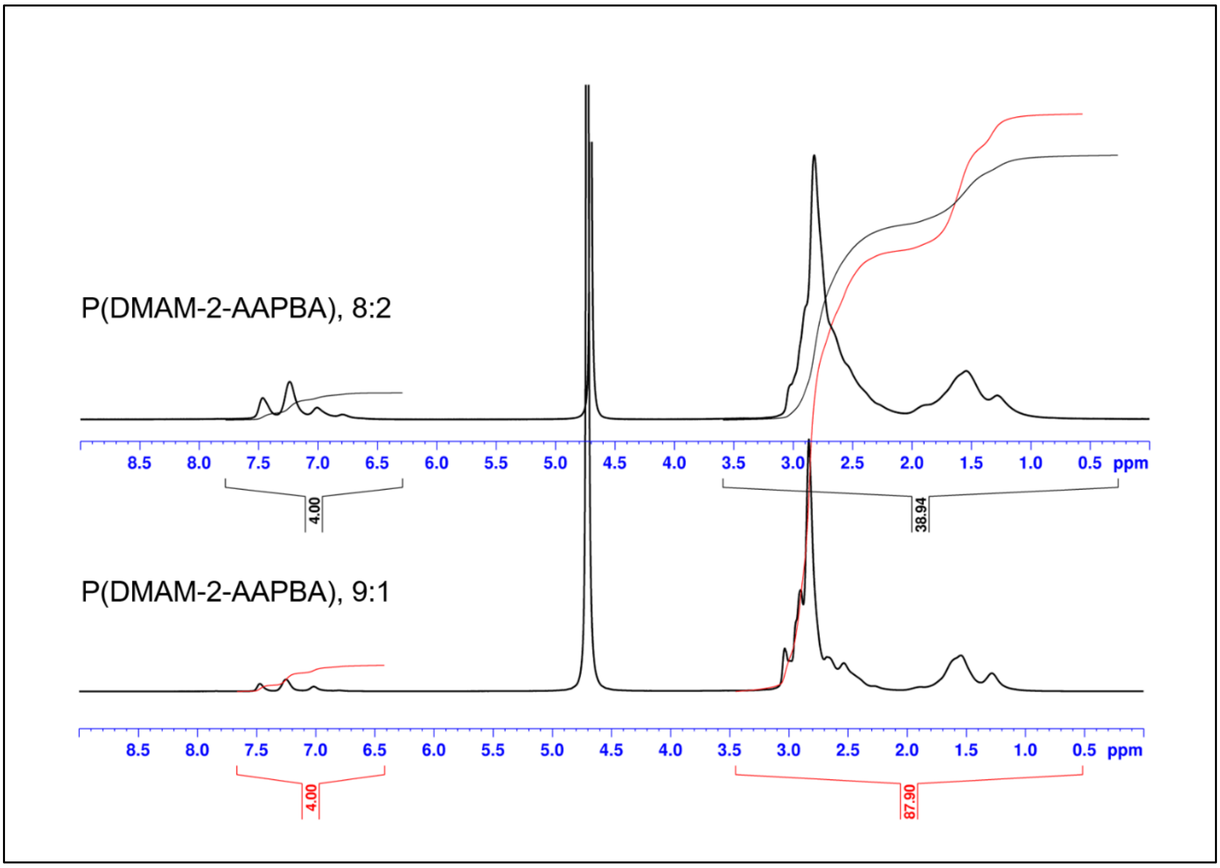

Figure S25. ^{1}H NMR spectra of DMAM-2-AAPBA copolymers (400 MHz D_2O, 295 K).

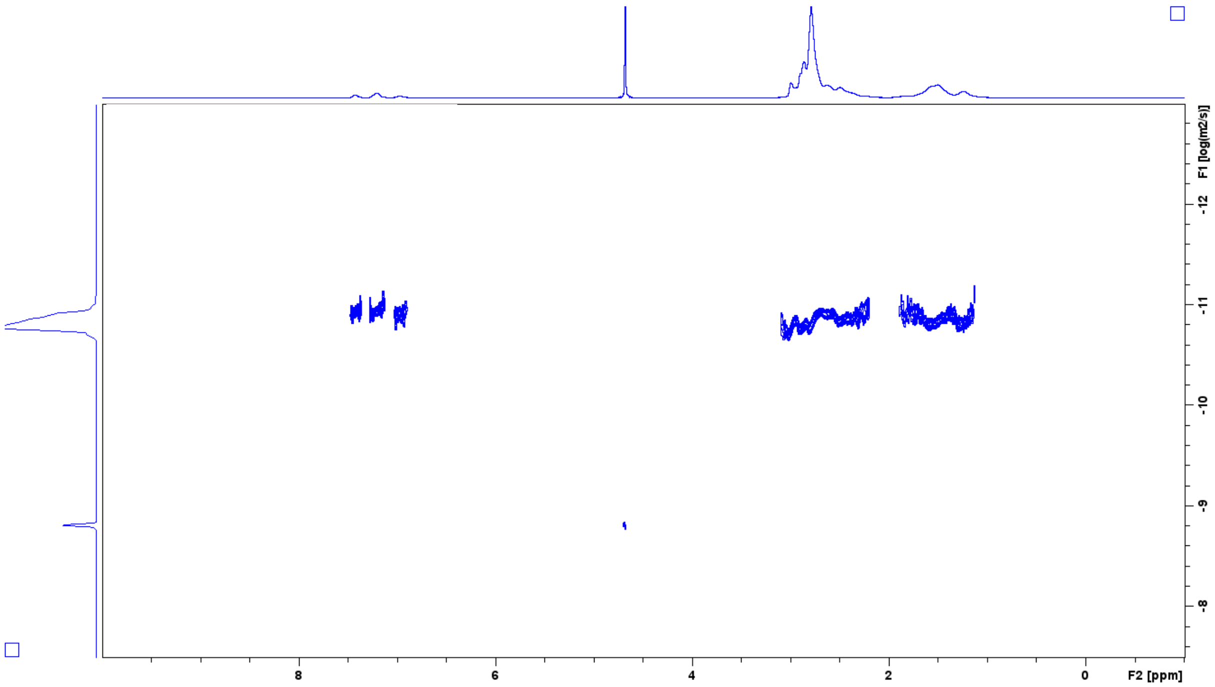

Figure S26. ^{1}H DOSY NMR spectrum of COP$^{9/1}$ (400 MHz D_2O, 295 K)..

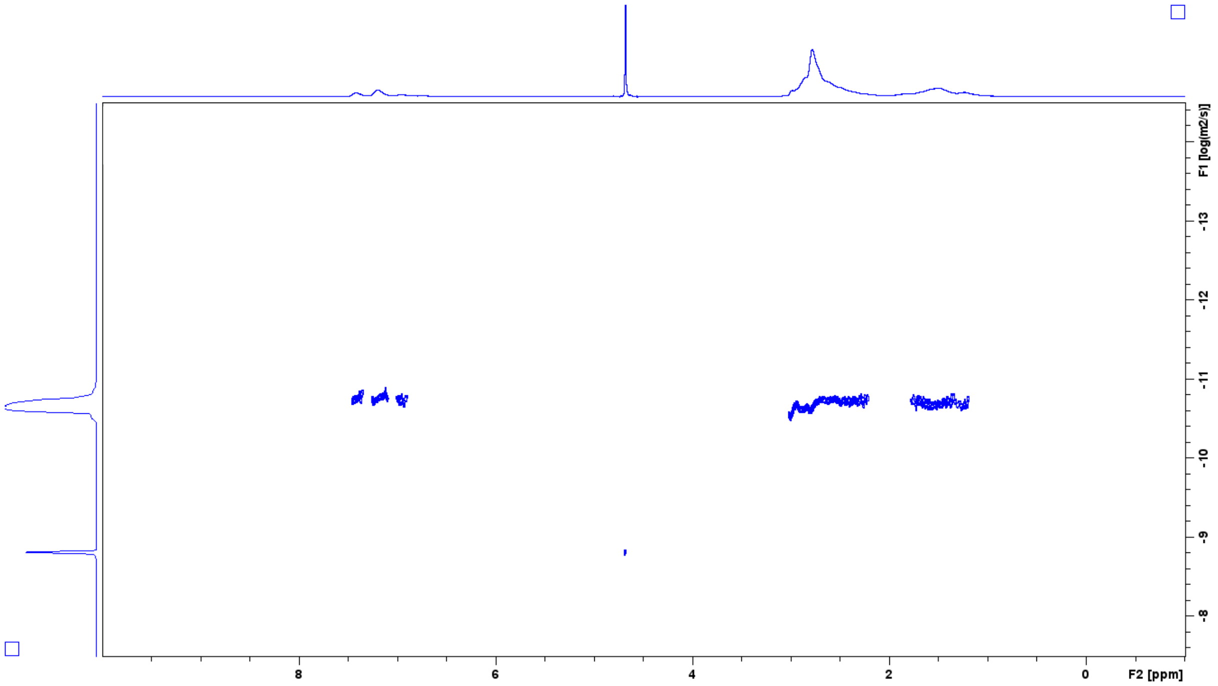

Figure S27. ^{1}H DOSY NMR spectrum of COP$^{8/2}$ (400 MHz D_2O, 295 K).

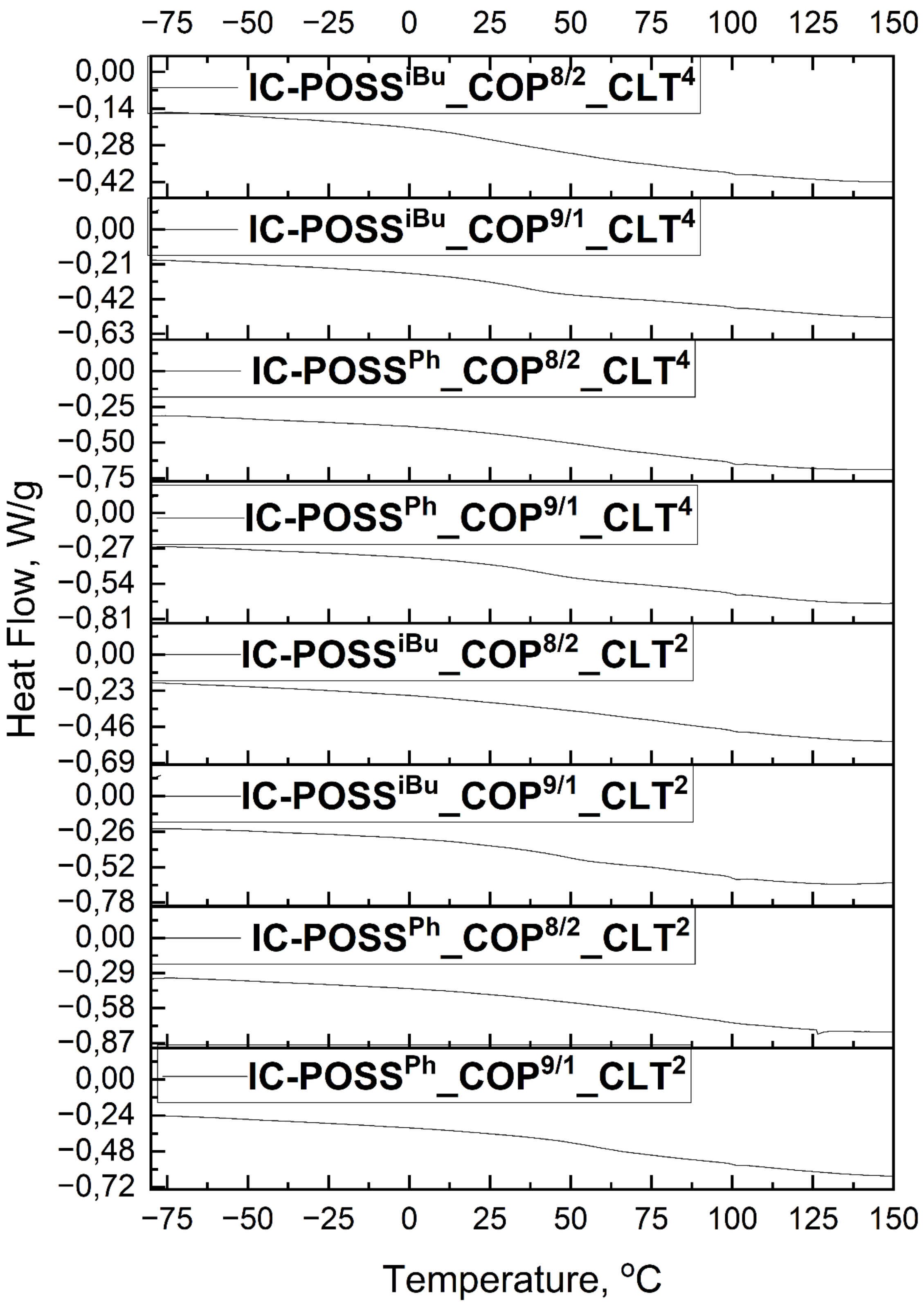

Figure S28. The DSC thermograms recorded for drug-loaded hybrid networks in the second heating.

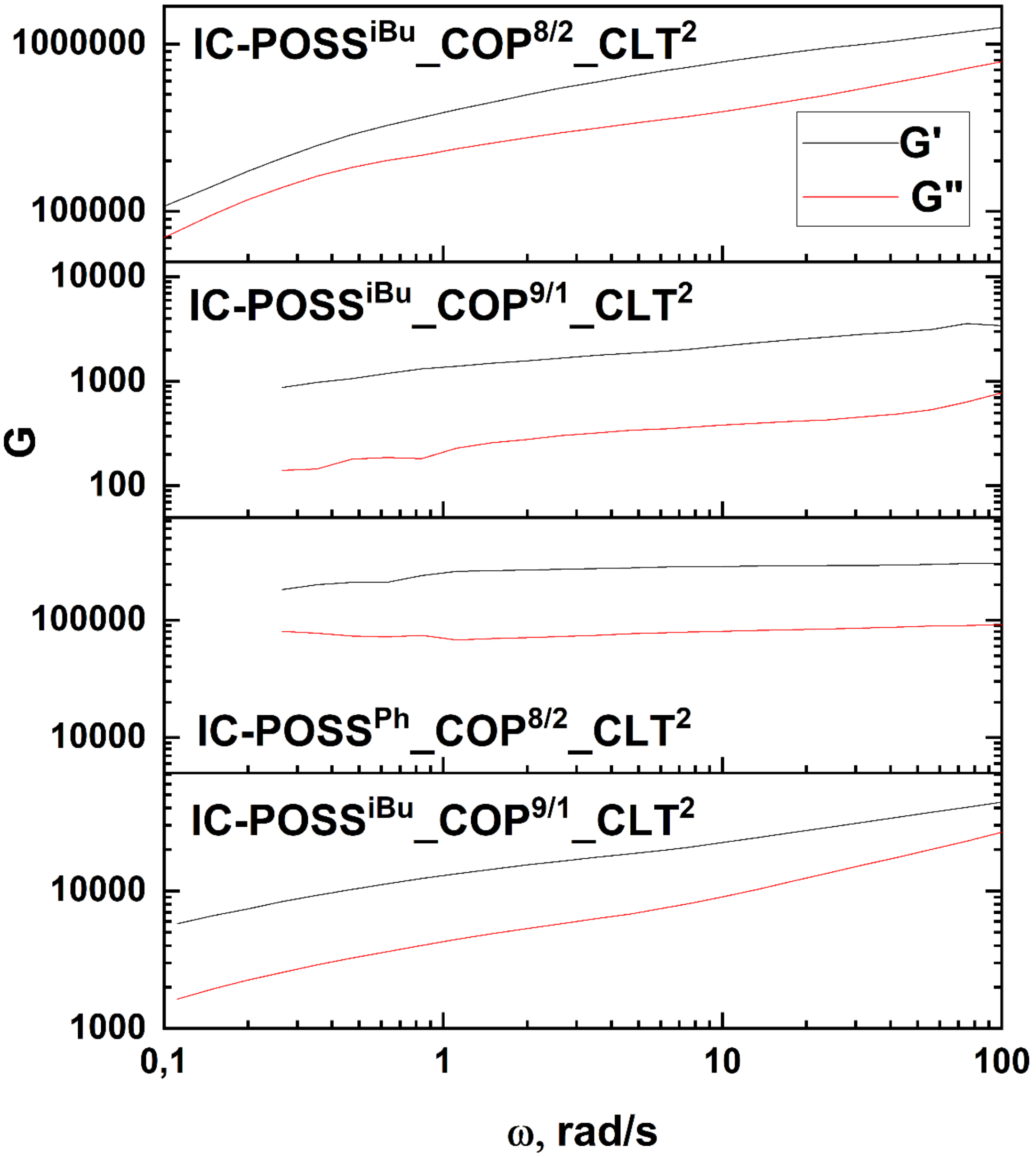

Figure S29. The frequency sweep tests performed for drug-loaded hydrated networks in which a molar ratio of drug per a cage was equal to 2:1.

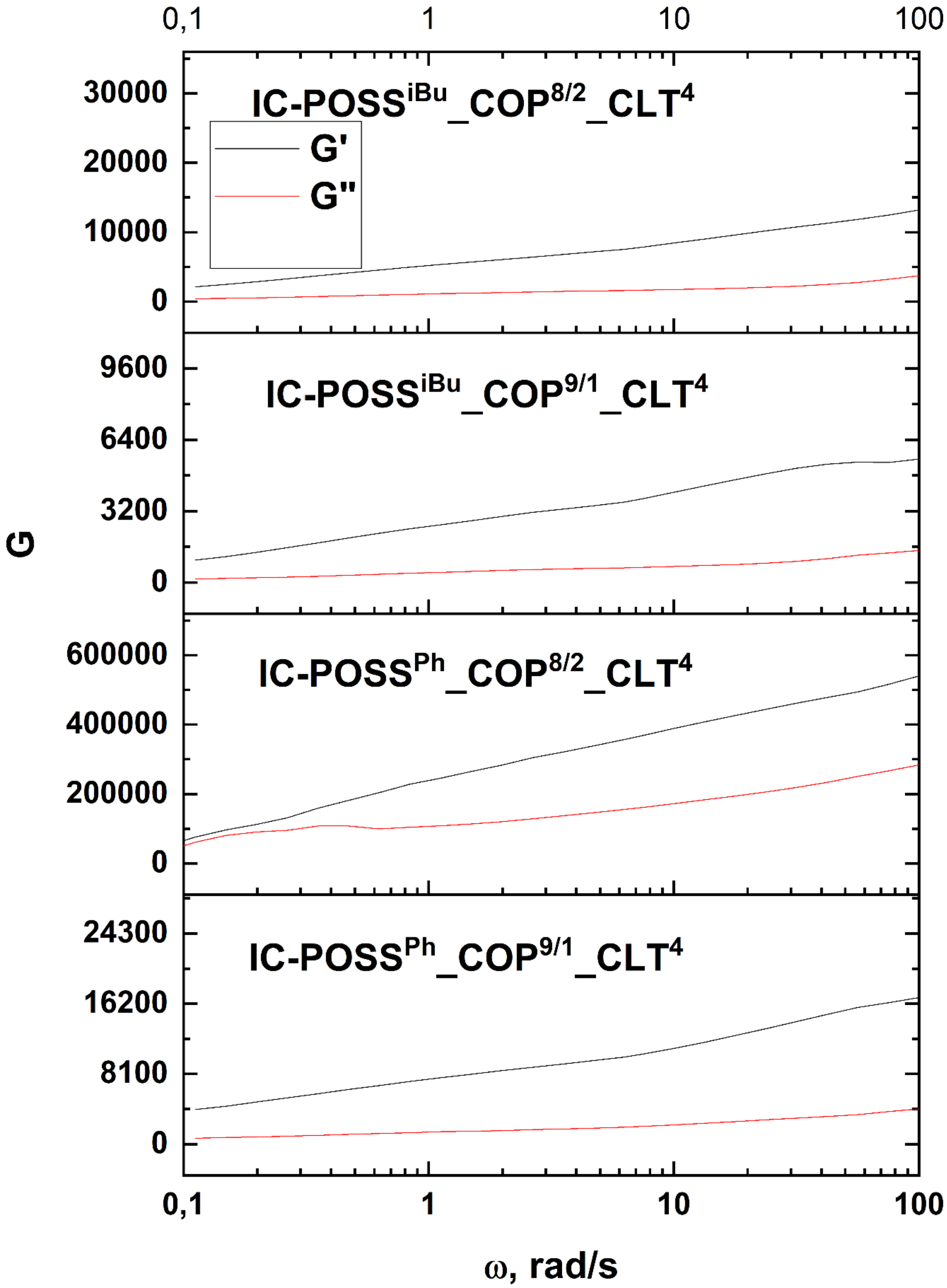

Figure S30. The frequency sweep tests performed for drug-loaded hydrated networks in which a molar ratio of drug per a cage was equal to 4:1.
